# Dynamic stiffness enables stage-specific properties mediating functional endothelialization on vascular implants

**DOI:** 10.1126/sciadv.adw8744

**Published:** 2026-07-29

**Authors:** Li Yang, Haoshuang Wu, Min He, Kaiyang Huang, Tiantian Zheng, Xia Yang, Rifang Luo, Yunbing Wang, Xingdong Zhang

**Affiliations:** ^1^ National Engineering Research Center for Biomaterials and College of Biomedical Engineering, Sichuan University, Chengdu 610065, China.; ^2^ Department of Critical Care Medicine, West China Hospital, Sichuan University, Chengdu 610041, China.; ^3^ Shanxi Provincial Key Laboratory for Functional Proteins, Shanxi Jinbo Bio-Pharmaceutical Co. Ltd, Taiyuan 030032, China.; ^4^ Tianfu Jincheng Laboratory (Frontier Medical Center), Chengdu 610213, China.

## Abstract

Achieving healthy, functional endothelialization represents the goal of neointima healing in cardiovascular implants but remains challenging due to strategies that fail to adapt dynamically to complex material-tissue interactions. We develop a biomimetic coating with dynamic stiffness via layer-by-layer assembly of recombinant humanized type III collagen and cystamine-modified hyaluronic acid, followed by postcrosslinking. The coating incorporates ECM-mimetic components and exhibits tunable stiffness that dynamically decreases in response to microenvironmental stimuli such as glutathione and reactive oxygen species. It exhibits stage-specific properties: initially suppressing acute thrombosis and directing cell adhesion; then facilitating a confluent, functional endothelial monolayer; driving smooth muscle cell contractile phenotype; and inducing anti-inflammatory macrophage polarization. In vivo, stents coated with our coating suppressed neointimal hyperplasia, achieving neointimal healing with morphology and function resembling native endothelium at 12 months. These findings reveal how dynamic stiffness guides material-tissue responses and provide a promising approach to enhance vascular implant healing.

## Teaser

A dynamic stiffness-tunable biomimetic coating guides vascular cells to promote endothelialization and implant healing.

## INTRODUCTION

Coronary artery disease (CAD), characterized by progressive atherosclerotic plaque accumulation in coronary arteries, represents the dominant cause of morbidity and mortality globally, regardless of country, ethnicity, or financial stratum (*1*, *2*). The vascular stent, implanted into narrowed vessels via guided balloon dilation, is viewed as the most effective intervention for treating CAD (*3*). Drug-eluting stents (DESs), which release antiproliferative agents such as paclitaxel or sirolimus, have notably reduced the incidence of in-stent restenosis (ISR) and are considered the clinical gold standard. However, the nonselective inhibition of smooth muscle and endothelial cell proliferation may impair reendothelialization, increasing the risk of late stent thrombosis (LST) and neointimal hyperplasia (NIH) (*4*). To mitigate these limitations, bioresorbable scaffolds (BRSs) were developed to provide temporary mechanical support and eliminate long-term foreign body presence (*5*, *6*). Nevertheless, by retaining the drug-eluting paradigm, BRSs still impair endothelial recovery, and polymer-based designs further face chronic inflammation and impaired vascular healing due to acidic degradation byproducts (*5*). These clinical limitations highlight the necessity of developing advanced surface modification strategies for BRSs that can appropriately regulate host responses during different healing stages.

Following stent implantation, a complex cascade of host responses occurs at the material-tissue interface. Early events include acute coagulation and inflammatory responses, which are closely related as they mutually induce and exacerbate each other (*7*, *8*). Sustained inflammation and impaired healing then promote smooth muscle cell (SMC) proliferation and excessive extracellular matrix (ECM) deposition, key drivers of ISR*.* These coupled processes collectively disrupt timely endothelialization and organized neointimal repair (*9*). In response, functional coatings had been developed to modulate cell-material interactions and promoted vascular regeneration. Most current approaches rely on biological or chemical cues, such as ECM-derived proteins (e.g., collagen, fibronectin, and gelatin), adhesive peptides (e.g., YIGSR and REDV), vascular endothelial growth factor (VEGF), and antibodies targeting endothelial progenitor cells (e.g., anti-CD34, CD133, and VEGFR2) (*10*–*12*). While these strategies enhance reendothelialization, they often neglect the role of physical microenvironmental cues in modulating cellular behavior.

The latest advances in mechanobiology have highlighted the direct and profound influence of biophysical cues, including matrix stiffness, micro/nano topography, and mechanical stretching, on various cell behaviors, such as adhesion, motility, differentiation, apoptosis, gene expression, and signal transduction (*13*–*18*). Among these, substrate stiffness has emerged as a particularly influential factor in cell growth and functional expression (*19*). For instance, stiff substrates are conducive to cell adhesion, spreading, migration, and proliferation but may also affect cellular phenotypes in a stiffness-dependent manner, leading to the loss of expression of particular phenotypic markers. In contrast, softer substrates tend to restrict these fundamental cellular behaviors but help preserve the native phenotype of certain cell types (*19*, *20*). During endothelium remodeling, substrate stiffness has been shown to modulate the formation of a confluent EC monolayer (*21*), influence the transition of SMC phenotype (*22*), and regulate macrophage polarization (*23*). Notably, different vascular cell types exhibit distinct sensitivities to mechanical stiffness, and healthy endothelial cells tend to develop on substrates with specific mechanical characteristics. These findings highlighted the potential of stiffness-mediated design to direct neointimal remodeling. However, existing approaches typically rely on static mechanical cues, which may be insufficient to accommodate the dynamically evolving microenvironment following stent implantation.

**Fig. 1.**
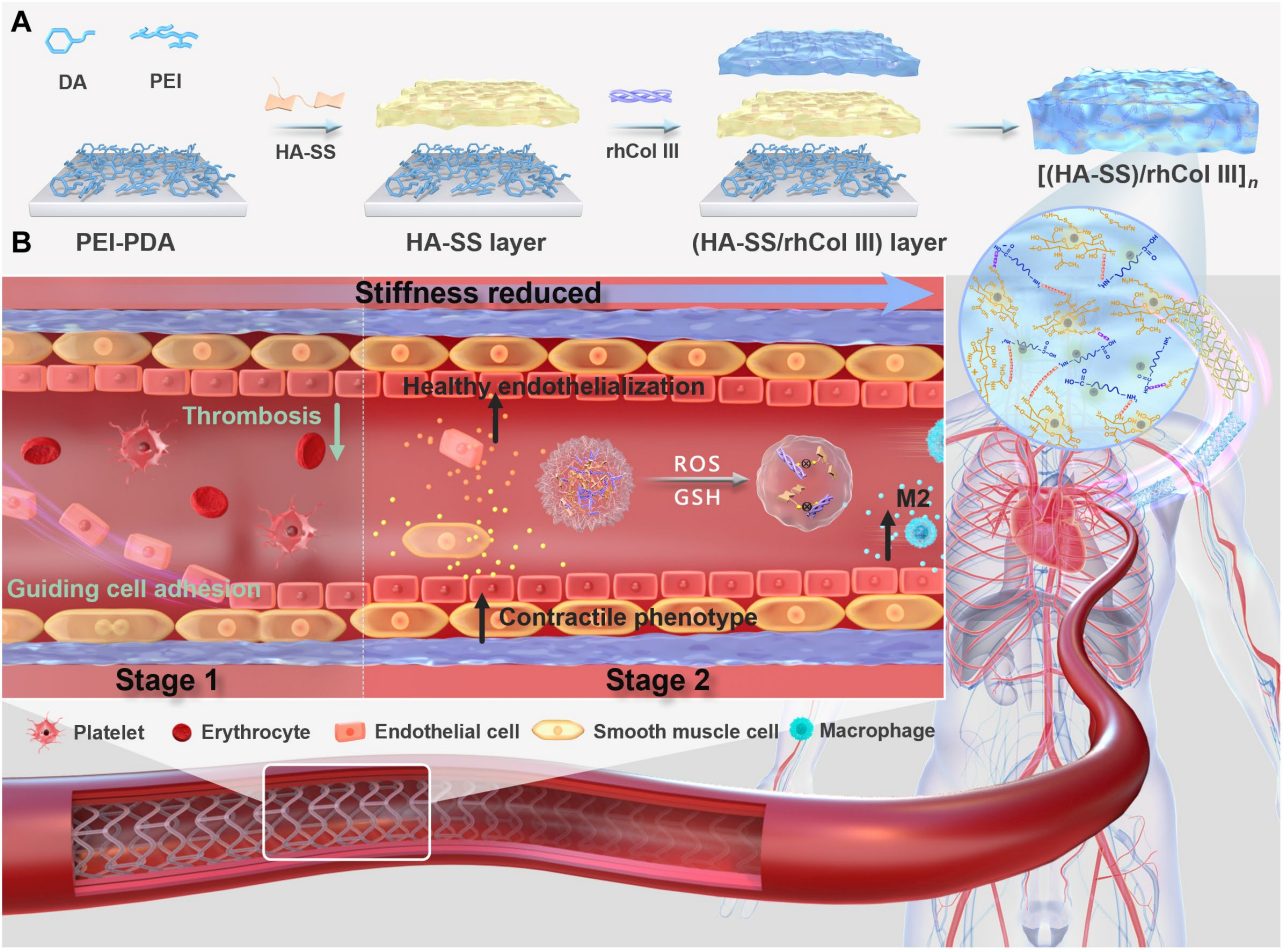
Development of a biomimetic coating with dynamic stiffness that exhibits stage-specific property for desired neointimal remodeling and functional endothelialization on a vascular stent. (**A**) Schematic of the [(HA-SS)/rhCol III]*_n_* fabrication process. Briefly, from bare stent to [(HA-SS)/rhCol III]*_n_*–coated stent, the preparation process initially involves amination, followed by the alternate deposition of cystamine-modified HA (HA-SS) and rhCol III via LBL assembly technology. The disulfide bond presented in HA-SS was ruptured after GSH and H_2_O_2_ treatments, resulting in the dynamic modulation of stiffness. (**B**) Illustration of the biomimetic coating platform with “stage-specific property” for desired neointimal remodeling and functional endothelialization. During the initial postimplantation period, the coating effectively suppressed acute thrombotic and directed the adhesion of vascular cells on the stent surface. Subsequently, reduced stiffness further selectively mediated vascular cell growth behaviors, which provided the basis for a vascular stent with the biomimetic coating to achieve ideal restoration of the native endothelium in vivo.

Given that, we put forward a biomimetic coating with dynamic stiffness that enables stage-specific properties. This coating is produced via layer-by-layer (LBL) assembly of tailored recombinant humanized type III collagen (rhCOL III) and cystamine-modified hyaluronic acid (HA-SS), followed by postcrosslinking. This biomimetic coating incorporates ECM-mimetic components and exhibits tunable and dynamic reductions in stiffness in response to stimuli from the microenvironment of the implantation site, such as glutathione and reactive oxygen species (ROS). This strategy effectively mediates vascular cell behavior in the complex local microenvironment after stent implantation, enabling functional endothelialization and desired neointimal healing on vascular devices, with the following advantages as detailed below: (i) The coating mimics ECM components by incorporating HA-SS and tailored rhCOL III, providing chemical cues with excellent biocompatibility. Notably, tailored rhCOL III is a novel biomaterial with low thrombogenicity and high cell affinity, designed by our team. (ii) The crosslinked coating exhibits a primary stiffness and can be dynamically regulated in response to the microenvironment at the implantation site, which is attributed to the rupture of disulfide bonds in HA-SS in the presence of glutathione (GSH) and ROS (Fig. 1A) (*24*). (iii) The obtained biomimetic coating presents stage-specific property after stent implantation, as described below: During the initial stage 1, the biomimetic coating with an optimized primary stiffness could effectively suppress acute thrombosis, and direct the adhesion of vascular cells. During the following stage 2 of vascular cell functionalization, the fracture of disulfide bonds was accompanied by a dynamic reduction in the stiffness of the coating, which in turn selectively mediated vascular cell growth behaviors, including the formation of confluent EC monolayers with high functional expression, the conversion of SMC to a contractile phenotype, and the anti-inflammatory polarization of macrophages (Fig. 1B). These two “stage-specific functions” lay a foundation for realizing optimal restoration and remodeling of the native-like endothelium.

The “dynamic stiffness” behavior and “stage-specific property” of this coating were thoroughly validated via ex vivo assays. Moreover, we investigated the safety and efficacy of the coating in vivo, and the results demonstrated the effectiveness of the coating in preventing intimal hyperplasia and even enabling desired neointimal remodeling resembling the native endothelium 12 months after implantation. These findings provide insights into how dynamic stiffness directs material and tissue response and offer a promising biomimetic approach to armor vascular implants for enhanced vascular tissue healing.

## RESULTS

### Fabrication and characterization of [(HA-SS)/rhCol III]*_n_*

In this study, biomimetic coatings [(HA-SS)/rhCol III]*_n_* with stiffness-responsive behavior were fabricated via the facile LBL self-assembly approach, where HA-SS represents cystamine-modified HA, rhCOL III refers to tailored recombinant humanized type III collagen, and *n* denotes the number of coating cycles. The corresponding formulation was shown in fig. S1. To ensure the feasibility of subsequent animal experiments, including soft tissue slices and immunohistochemistry, poly (l-lactic acid) (PLA; sheets and stents) substrates were selected as template materials. The HA-SS was first synthesized, in which the disulfide bonds could be ruptured in response to GSH and ROS present in the blood microenvironment. To coat the PLA substrates with [(HA-SS)/rhCol III]*_n_*, we first modified the bare PLA surface using dopamine (PDA) and polyethyleneimine (PEI) to generate an amine-rich primer (PEI-PDA) to provide further reaction sites. The PEI-PDA–modified substrates were then alternately immersed in HA-SS and rhCol III solutions to complete one cycle. Following several cycles, the above-mentioned samples were immediately immersed in MES buffer containing 1-ethyl-3-(3-dimethylaminopropyl)carbodiimide (EDC) and *N*-hydroxysuccinimide (NHS) to obtain tough [(HA-SS)/rhCol III]*_n_* coatings via the covalent crosslinking of HA-SS and rhCol III. The obtained samples were denoted as [(HA-SS)/rhCol III]*_n_*. To verify the “dynamic stiffness” properties of our [(HA-SS)/rhCol III]*_n_* coatings, 1, 6-diaminohexane dihydrochloride, which has a similar structure to cystamine dihydrochloride but lacks redox responsiveness, was selected to synthesize HA-CC for comparison with HA-SS. The ((HA-CC)/rhCol III)*_n_* coatings were produced in a manner similar to the response ones.

**Fig. 2.**
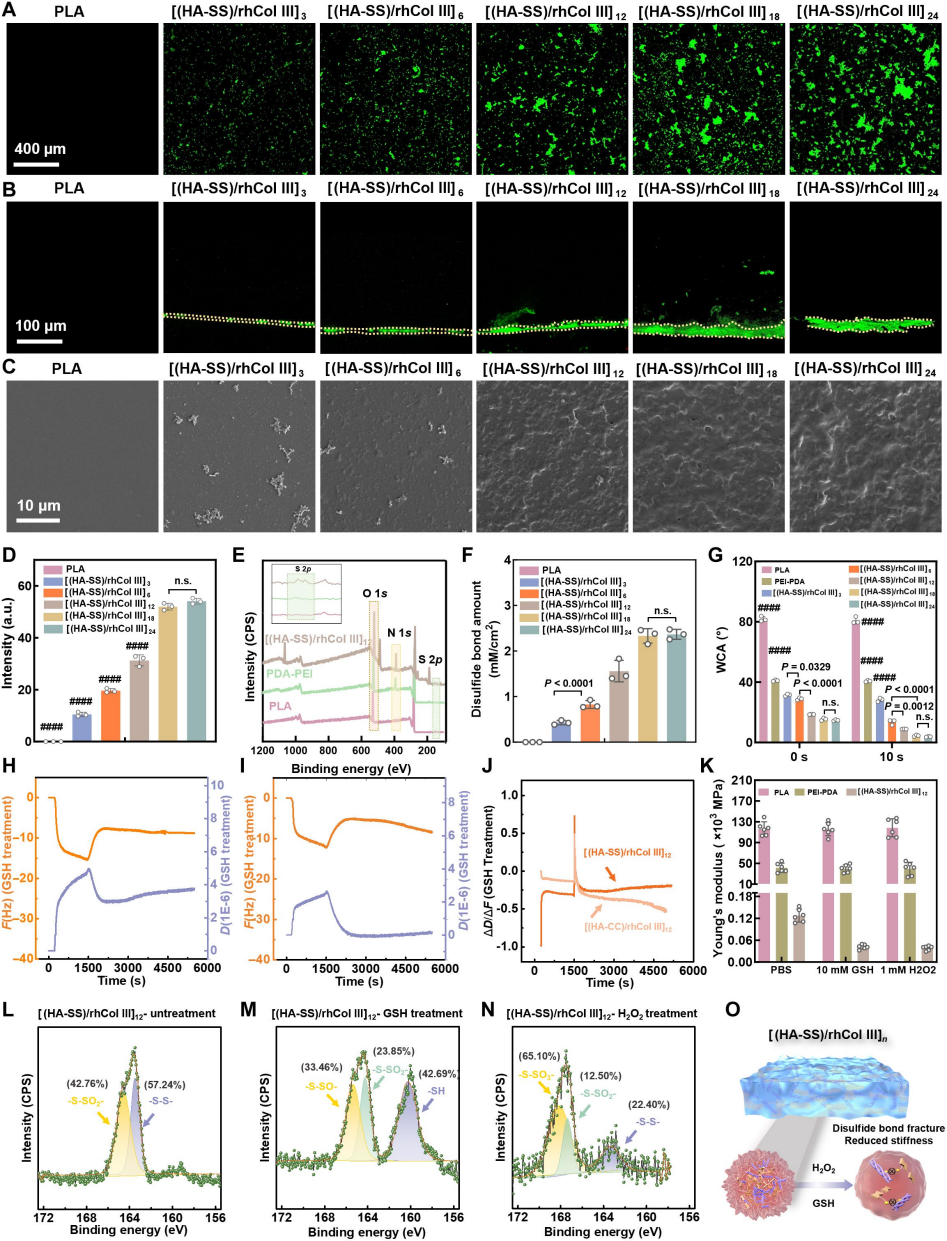
Characterization of [(HA-SS)/rhCol III]*_n_* and ((HA-CC)/rhCol III)*_n_* coatings. Representative (**A**), horizontal surface and (**B**) vertical section fluorescent images of [(HA-SS)/rhCol III]*_n_*, investigated by confocal laser scanning microscope (CLSM). Scale bars, 400 and 100 μm. (**C**) Representative SEM images of [(HA-SS)/rhCol III]*_n_*. Scale bar, 10 μm. (**D**) Corresponding quantification of fluorescence intensity from images as in (A) (*n* = 3 independent samples). (**E**) Wide-scan XPS spectra and local enlarged images from wide-scan XPS spectra of uncoated, PDA-PEI–, and [(HA-SS)/rhCol III]_12_–coated PLA sheets. (**F**) Disulfide bond amount on the surface of different samples, measured by DTNB assay (*n* = 3 independent samples). (**G**) WCA of different samples (*n* = 3 independent samples). Representative real-time QCM-D monitoring of the variation of *F* and *D* values of (**H**) [(HA-SS)/rhCol III]_12_ and (**I**) ((HA-CC)/rhCol III)_12_ under the treatment with GSH. (**J**) Representative Δ*D*/Δ*F* curve of [(HA-SS)/rhCol III]_12_ and ((HA-CC)/rhCol III)_12_ layers treated with GSH. (**K**) Young’s modulus of PLA, PEI-PDA, and [(HA-SS)/rhCol III]*_n_* groups untreated and treated with GSH and H_2_O_2_, respectively, observed by AFM (*n* = 6 independent samples). Corresponding high-resolution S2*p* spectrum from wide scan XPS spectra of [(HA-SS)/rhCol III]_12_ group (**L**) without treatment and with (**M**) GSH and (**N**) H_2_O_2_ treatment. (**O**) Schematic illustration of the disulfide bonds in [(HA-SS)/rhCol III]*_n_* fracturing under GSH and H_2_O_2_ stimulation, resulting in a decrease in stiffness. One-way analysis of variance (ANOVA) was used for the comparisons in (D) and (E). Two-way ANOVA was used for the comparisons in (F). All error bars are means ± SD (*P* values < 0.05 were considered statistically significant, and #### indicated *P* values < 0.0001 compared with other groups). n.s., not significant.

The ^1^ H nuclear magnetic resonance (NMR) spectra confirmed the synthesis of HA-SS and HA-CC (fig. S2), as evidenced by the presence of new peaks at ∼2.9 and 3.3 parts per million (ppm) for HA-SS and 1 to 2 ppm for HA-CC in comparison to HA, which was consistent with other studies (*25*). The successful fabrication of [(HA-SS)/rhCol III]*_n_* was visualized directly via the fluorescence signal of fluorescein isothiocyanate (FITC)–labeled rhCol III, as shown in Fig. 2 (A and B). Scanning electron microscopy (SEM) results revealed that the [(HA-SS)/rhCol III]*_n_* surfaces progressively became more homogeneous as the number of layers increased. This was attributed to the organized deposition of abundant ECM-mimetic components of HA-SS and rhCol III (Fig. 2C). The fluorescence signal on the surface of the [(HA-SS)/rhCol III]*_n_* also increased stepwise from 10.5 to 54.1% as the number of layers increased from 3 to 24 (Fig. 2, A and D). Meanwhile, the thickness of [(HA-SS)/rhCol III]*_n_* increased gradually from 0.667 μm [for the [(HA-SS)/rhCol III]_3_ group] to 2.333 μm [for the [(HA-SS)/rhCol III]_24_ group] (Fig. 2B and fig. S3). To further confirm the successful immobilization of HA-SS and rhCol III, the determinations of x-ray photoelectron spectroscopy (XPS) and reflection-absorption fourier transform infrared spectroscopy (RA-FTIR) were carried out. The apparent presence of N 1*s* peaks and S 2*p* on the PEI-PDA and [(HA-SS)/rhCol III]*_n_* (*n* = 3, 6, 12, 18, and 24) groups indicated the that PDA-PEI coating and HA-SS were immobilized (Fig. 2E and fig. S4A). The successful fabrication of the [(HA-SS)/rhCol III]*_n_* coatings was further confirmed by the C 1*s* high-resolution fits of XPS data and RA-FTIR results (figs. S4, B to H, and S5, A and B; see the Supplementary Materials for detailed discussion) (*26*, *27*). We then quantified the amount of disulfide bonds in the [(HA-SS)/rhCol III]*_n_* coatings and found that it ranged from 0.43 to 2.37 mM/cm^2^ as the number of layers increased from 3 to 24 (Fig. 2F). The substantial change in water contact angle (WCA) in the PLA after modification of PEI-PDA and [(HA-SS)/rhCol III]*_n_* indicated effective functionalization (Fig. 2G). In particular, the abundance of amino groups in the PEI-PDA improved the hydrophilic properties of the PLA, resulting in a WCA of 40.7°. The [(HA-SS)/rhCol III]*_n_* coatings became more hydrophilic as the coating cycles increased from 3 to 24, with the WCA decreasing from 28.5° to 14.7°. Notably, the [(HA-SS)/rhCol III]*_n_* (*n* = 18 and 24) coatings could rapidly form a hydrated layer and thus exhibit superhydrophilic properties as evidenced by a rapid drop in WCA value to nearly 0° after 10 s (fig. S6). In total, those signals confirmed the effective of [(HA-SS)/rhCol III]*_n_* coating on the PLA sheets.

The real-time assembly process of [(HA-SS)/rhCol III]*_n_* was investigated by quartz crystal microbalance with dissipation (QCM-D). Specifically, the adsorbed mass is represented by the frequency shift (Δ*F*) (fig. S7A), and the viscoelastic properties of the adsorbate are evaluated by the dissipation change (Δ*D*) (fig. S7B). The changes in the Δ*D*/Δ*F* values are closely correlated with the soft and stiff states of the adsorbents, with higher values indicating a softer adsorbed layer (*28*). QCM-D results demonstrated a gradual increase in Δ*F* and Δ*D* value as the number of layers increased, which evidenced the successful adsorption of the coating components (figs. S8 and S9). To confirm the dynamic stiffness properties in response to external stimuli, we selected [(HA-SS)/rhCol III]_12_ and ((HA-CC)/rhCol III)_12_ as research objects and further investigated their behavior under ROS and GSH stimuli. With the addition of H_2_O_2_, the *F* values of [(HA-SS)/rhCol III]_12_ were progressively increased as a result of disulfide bond rupture and the washing away of coating components by the fluid (fig. S10A). Nevertheless, the *F* values of [(HA-SS)/rhCol III]_12_ were first decreased and then gradually increased under GSH-stimulated conditions (Fig. 2H). This phenomenon was due to the reaction between GSH, a polypeptide with both amine and carboxyl groups, and the amino group in the coatings (*29*). In contrast to [(HA-SS)/rhCol III]_12_, dissipation variations of ((HA-CC)/rhCol III)_12_ were much smaller and even virtually unchanged in later stages with GSH and H_2_O_2_ treatments (Fig. 2I and fig. S10B). Next, we calculated the Δ*D*/Δ*F* values by analyzing the *F*-time and *D*-time curves. The Δ*D*/Δ*F* values of [(HA-SS)/rhCol III]_12_ gradually increased with both GSH and H_2_O_2_ treatments, indicating a softening of the coating (Fig. 2J and fig. S10C). In contrast, the ((HA-CC)/rhCol III)_12_ group showed no obvious change with H_2_O_2_ treatment and even a gradual decrease with GSH treatment. This strongly supported the notion that the [(HA-SS)/rhCol III]_12_ displays stiffness response characteristics. Last, we quantitatively evaluated the stiffness variations of the biomimetic coatings in phosphate-buffered saline (PBS), GSH, and H_2_O_2_. The Young’s modulus of [(HA-SS)/rhCol III]*_n_* coatings (*n* = 3 to 24) ranged from 28.6 × 10^3^ to 32 kPa in PBS, 20.4 × 10^3^ to 24 kPa in GSH, and 16.7 × 10^3^ to 22 kPa in H_2_O_2_, respectively. These results demonstrated the dynamic stiffness of the [(HA-SS)/rhCol III]*_n_* coatings upon treatment with GSH and H_2_O_2_ (Fig. 2K). For comparison, the ((HA-CC)/rhCol III)*_n_* coatings subjected to the above-described treatments showed no dynamic stiffness characteristic, as evidenced by no difference in the variation range of Young’s modulus (fig. S11).

Next, we selected [(HA-SS)/rhCol III]*_n_* (*n* = 6, 12, and 18) and further investigated the changes in disulfide bonding under redox conditions by analyzing the S 2*p* high-resolution data before and after treatment with GSH and H_2_O_2_ (Fig. 2, L to N, and fig. S12, A to C). In the untreated [(HA-SS)/rhCol III]*_n_* groups, two peaks were observed at 163.4 eV (−S─S−) and 164.5 eV (−S─SO_2_−), indicative of slight disulfide bond oxidation. After GSH treatment, three peaks emerged at 160.6 eV (-SH), 165.8 eV (−S─SO−), and 164.5 eV (−S─SO_2_−), corresponding to disulfide bond reduction. In contrast, H_2_O_2_-treated groups exhibited three peaks at 163.4 eV (−S─S−), 167.8 eV (−S─SO_2_−), and 168.1 eV (−S─SO_3_−) (*30*), reflecting further oxidation of the disulfide bonds (*29*).

Together, these experimental results demonstrated that the stiffness of [(HA-SS)/rhCol III]*_n_* coatings gradually decreased from 28.6 × 10^3^ kPa to 32 kPa as the number of layers increased from 3 to 24 (Fig. 2O). In particular, the stiffness of the [(HA-SS)/rhCol III]*_n_* coatings (*n* = 18 and 24) treated with GSH and H_2_O_2_ was within the range healthy vascular artery stiffness (between 20 and 40 kPa) (*31*), which provided a platform for subsequent assessment of the effects of a native ECM-like physical stiffness environment on cellular growth behavior. Given the relatively higher levels of GSH in healthy human blood compared to H_2_O_2_ (*32*, *33*), we subsequently focused on the impact of the GSH-induced dynamic stiffness of the coatings on biological function while deprioritizing further investigation of H_2_O_2_ treatment. Such a strategy not only mimicked the cellular living environment in terms of mechanical property but also established the basis for the subsequent development of multiple biological functions by the loading of the ECM components HA and rhCol III.

### In vitro and ex vivo antithrombogenicity

**Fig. 3.**
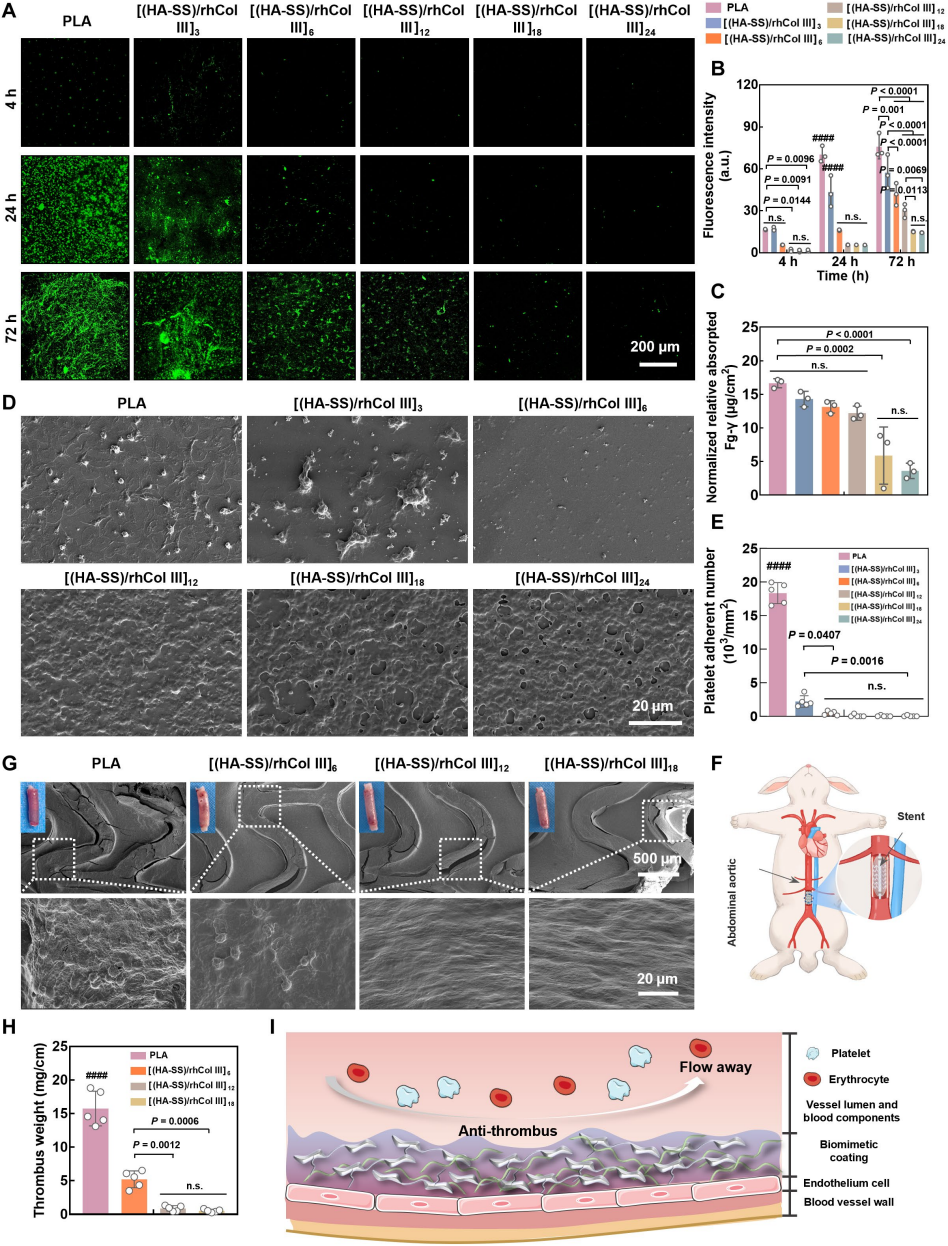
In vitro and in vivo hemocompatibility of the [(HA-SS)/rhCol III]*_n_* coatings (*n* = 3, 6, 12, 18, and 24). (**A**) Representative fluorescence images showing the improved capacity of the [(HA-SS)/rhCol III]*_n_* coatings in suppressing FITC-hFg adhesion with enhanced hydrophilicity. Scale bar, 200 μm. (**B**) Corresponding quantification of fluorescence intensity from images as in (A) (*n* = 3 independent samples). (**C**) Normalized relative absorbed Fg-γ amounts for different samples after 24 hours of incubation (*n* = 3 independent samples). (**D**) Representative platelets adhered on the surfaces of uncoated and [(HA-SS)/rhCol III]*_n_*–coated PLA sheets, observed by SEM. Scale bar, 10 μm. (**E**) Corresponding quantification of platelets adherent number from images as in (D) (*n* = 5 independent samples). (**F**) Schematic illustration for stent implantation into the abdominal aorta of New Zealand White rabbits. (**G**) SEM images showing the thrombus formation on the bare PLA stent and the [(HA-SS)/rhCol III]*_n_*–coated stents (*n* = 6, 12, and 18) in a rabbit model. Scale bars, 500 and 20 μm. (**H**) Corresponding quantified data of platelet adherent number from the SEM images as in (G) (*n* = 5 independent samples in independent animals). (**I**) Schematic illustrating the anti-thrombotic behavior of the [(HA-SS)/rhCol III]*_n_* coatings. Created in BioRender. Wu, H. (2026) https://BioRender.com/syb4ne0. One-way ANOVA was used for the comparisons in (C), (E), and (H). Two-way ANOVA was used for the comparisons in (B). All error bars are means ± SD (*P* values < 0.05 were considered statistically significant, and #### indicated *P* values < 0.0001 compared with other groups).

Following balloon dilatation and intravascular implantation, fibrinogen rapidly adsorbs onto the surface of the materials, promoting platelet adhesion and activation at the deendothelialized vessel wall (*34*, *35*). Over time, the denatured fibrin networks the activated platelets, leading to the eventual formation of a thrombus. Given the antithrombotic properties of tailored rhCol III as demonstrated in our previous studies (*28*), it is believed that the [(HA-SS)/rhCol III]*_n_* coatings could develop anticoagulant potency upon loading with adequate amounts of rhCol III. To corroborate this, we performed the FITC-labeled human fibrinogen (FITC-hFg) adsorption test on our [(HA-SS)/rhCol III]*_n_* coatings for 4 hours, 1 day, and 3 days, respectively. The results demonstrated notably higher fibrinogen adhesion on bare PLA surfaces compared to the [(HA-SS)/rhCol III]*_n_* groups at any designated time points. For the [(HA-SS)/rhCol III]*_n_* groups, the fluorescence intensity progressively decreased with the increasing number of layers. Notably, almost no fluorescence signal was observed on the superhydrophilic [(HA-SS)/rhCol III]_18_ and [(HA-SS)/rhCol III]_24_ surfaces, consistent with previous studies reported that the hydration layer on the superhydrophilic surfaces was effective in suppressing fibrinogen adhesion (Fig. 3A) (*36*). Quantitative analyses revealed a considerable increase in fluorescence intensity for the bare PLA and [(HA-SS)/rhCol III]*_n_* groups (*n* = 3, 6, and 12) over time, from 4 hours to 3 days [e.g., 16.6 to 75.8% for PLA, 17.1 to 65.6% for [(HA-SS)/rhCol III]_3_, 5.7 to 41.9% for [(HA-SS)/rhCol III]_6_, and 2.1 to 30.1% for [(HA-SS)/rhCol III]_12_]. In contrast, no pronounced changes were observed in the [(HA-SS)/rhCol III]*_n_* (*n* = 18 and 24) groups, with almost no detectable fluorescent signals (Fig. 3B). Thereafter, the conformational changes of the adsorbed fibrinogen were detected by fibrinogen γ-chain (Fg-γ) enzyme-linked immunosorbent assay (ELISA) kits. Compared with the control bare PLA, [(HA-SS)/rhCol III]*_n_* coatings facilitated the natural conformation maintenance of the adsorbed proteins, especially in [(HA-SS)/rhCol III]*_n_* (*n* = 18 and 24), as evidenced by fewer adsorbed Fg-γ (Fig. 3C). Collectively, the [(HA-SS)/rhCol III]*_n_* (*n* = 18 and 24) coatings notably suppressed nonspecific adsorption and denaturation of plasma proteins, a critical factor in ensuring good hemocompatibility during the initial stages following stent implantation.

To evaluate the potential of our [(HA-SS)/rhCol III]*_n_* coatings to suppress clot formation, we carried out in vitro platelet adhesion assay using platelet-rich plasma (PRP) (fig. S13) from healthy human volunteers. After 2 hours of incubation, a notable number of highly activated platelets adhered and aggregated on the surface of the PLA group, presenting flattened morphology with pseudopodia spreading. A considerable number of aggregated platelets with interwoven pseudopodia still adhered to the surface of [(HA-SS)/rhCol III]_3_; however, with the increase in the number of layers, almost no platelets were observed on the surface of [(HA-SS)/rhCol III]*_n_* (*n* = 12, 18, and 24) (Fig. 3, D and E). Consistent with the above results, ex vivo arteriovenous (AV) shunt assay using New Zealand white rabbit further confirmed that [(HA-SS)/rhCol III]*_n_* (*n* = 12, 18, and 24) has excellent antithrombotic properties (fig. S14, A to D; see the Supplementary Materials for detailed discussion) (*37*).

Encouraged by the above results, we further examined whether our [(HA-SS)/rhCol III]*_n_* coatings would function on vascular stents (Fig. 3F). As expected, similar trends were observed as described above for the in vitro platelet adhesion and ex vivo AV shunt assays. Severe thrombi were detected on the bare PLA stent, evidenced by thick aggregations of erythrocytes interwoven with dense filaments. Excitingly, when the (HA-SS)/rhCol III coating was applied in more than 12 cycles, almost no thrombi or even no platelets were observed on the [(HA-SS)/rhCol III]*_n_* stents (*n* = 12 and 18) (Fig. 3G). The quantitative analysis confirmed that the [(HA-SS)/rhCol III]_18_ coating demonstrated optimal antithrombotic performance, with negligible platelet adhesion and minimal thrombosis (Fig. 3H).

Together, [(HA-SS)/rhCol III]*_n_* coatings (*n* ≥ 18) with superhydrophilic properties showed superior performance in preventing nonspecific adsorption and denaturation of plasma proteins. The ex vivo and in vivo hemocompatibility assays (Fig. 3, D to H) demonstrated that even for the [(HA-SS)/rhCol III]*_n_* coatings (*n* = 6 and 12) without superhydrophilic properties, they still could effectively inhibit platelet adhesion and activation, retard the process of blood coagulation, or even completely inhibit it. Therefore, it was reasonable to conclude that the favored hemocompatibility of [(HA-SS)/rhCol III]*_n_* was attributed to the synergistic effects of the superhydrophilicity that mediated resistance to the adsorption and denaturation of plasma proteins and the inherent antithrombotic properties of rhCOL III (Fig. 3I). In total, our [(HA-SS)/rhCol III]*_n_* strategy was expected to show superiority in clinical application.

### Effects of the [(HA-SS)/rhCol III]*_n_* on HUVECs behaviors in vitro

**Fig. 4.**
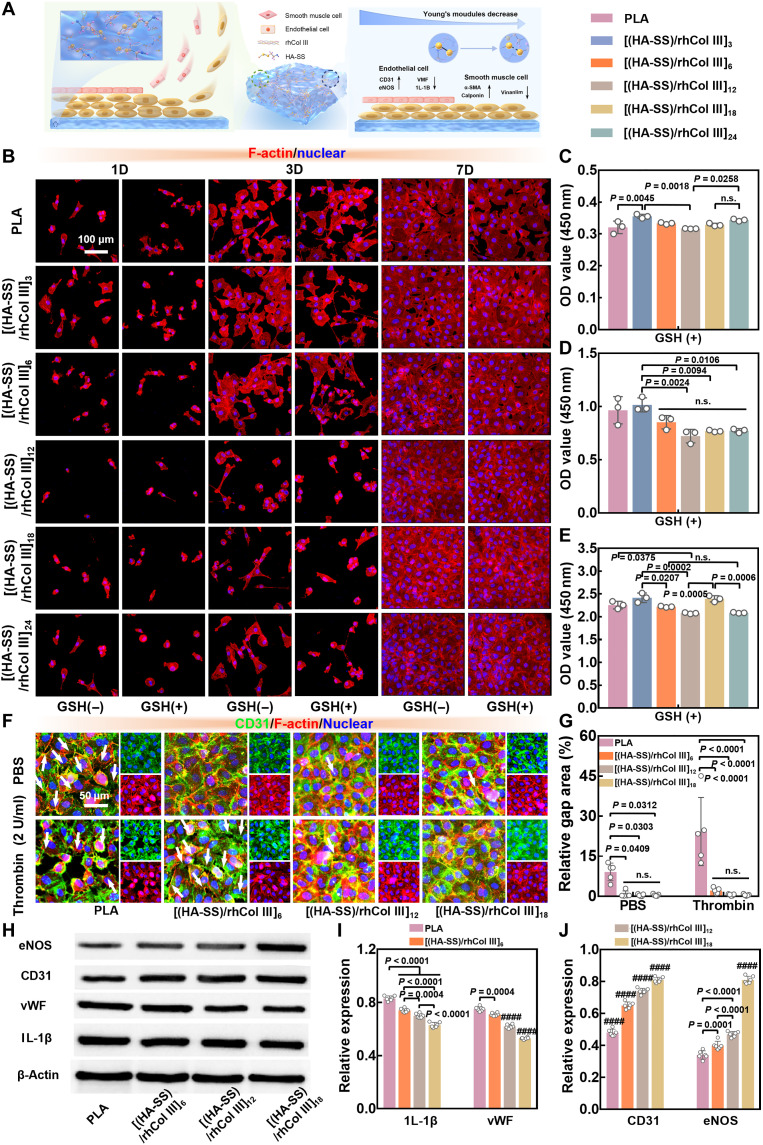
Effects of the [(HA-SS)/rhCol III]*_n_* on cell morphology and functions of HUVECs in vitro. (**A**) Schematic of the samples cultured with HUVECs treated by PBS (GSH−) and glutathione (GSH+) for 1, 3, and 7 days, respectively. The stiffness of the [(HA-SS)/rhCol III]*_n_* coatings decreased with the addition of GSH, demonstrating adverse cell adhesion and proliferation but favoring the maintenance of high levels of endothelial function. (**B**) Representative fluorescence images showing the adhesion and proliferation of HUVECs were synergistically regulated by biophysical cues (stiffness) and biochemical cues (rhCol III amount). Scale bar, 100 μm. Cell viability of HUVECs cultured on uncoated and [(HA-SS)/rhCol III]*_n_*–coated PLA sheets (*n* = 3, 6, 12, 18, and 24) with GSH treatment after (**C**) 1 day, (**D**) 3 days, and (**E**) 7 days of culture (*n* = 3 independent samples). (**F**) Representative immunofluorescence images of monolayer integrity of HUVECs formed on PLA and [(HA-SS)/rhCol III]*_n_* (*n* = 6, 12, and 18) before and after treatment with thrombin (2 U/ml). (**G**) Corresponding quantification of gap area from images as in (F) (*n* = 3 independent samples). (**H**) Typical Western blot bands and corresponding relative quantified data of (**I**) and (**J**) IL-1β, vWF, CD31, and eNOS proteins (*n* = 6 independent samples). One-way ANOVA was used for the comparisons in (C) to (E). Two-way ANOVA was used for the comparisons in (G), (I), and (J). All error bars are means ± SD (*P* values < 0.05 were considered statistically significant, and #### indicated *P* values < 0.0001 compared with other groups). OD, optical density.

Upon contact with foreign implants, cell adhesion is the first cellular event that occurs and strongly influences subsequent cellular behaviors, such as migration and proliferation. In addition to biochemical cues, cells can sense physical cues such as changes in substrate stiffness and translate these stimuli into biochemical signals, either individually or synergistically (with some biochemical factors) modulating various aspects of cellular behavior, which contribute to the control of cell fate (*38*). Here, we provided insights into the synergetic influence of biochemical and biophysical cues on endothelium repair by conducting an adhesion test of human umbilical vein endothelial cells (HUVECs) on [(HA-SS)/rhCol III]*_n_* coatings in the medium supplemented with 30 μM GSH (Fig. 4A). The immunofluorescence staining results (Fig. 4B) showed that within the first 3 days, the density and coverage of HUVECs that adhered to relatively stiff substrates, such as PLA and [(HA-SS)/rhCol III]*_n_* (*n* = 3 and 6), were higher compared to those on softer substrates [(HA-SS)/rhCol III]*_n_* (*n* = 12, 18, and 24), with or without GSH treatment. Nevertheless, after 1 week of incubation, more HUVECs with spindle morphology and well-spreading were observed on soft substrates and proliferated into confluent monolayers, irrespective of GSH addition. On the contrary, HUVECs attached to the stiff substrates were poorly connected and developed small pores, implying a dysfunctional endothelial barrier (*39*). Notably, cell proliferation was suppressed in groups with GSH treatment compared to those without GSH treatment at any designated time point, consistent with reports that lower stiffness surfaces are unfavorable for cell adhesion and proliferation (Fig. 4, C to E, and fig. S15, A to C) (*40*). Most impressively, HUVEC growth behavior was influenced not merely by a single biophysical or biochemical cue, but by their collaborative efforts. This was evidenced by the fact that cell adhesion and proliferation were progressively suppressed with the decreased stiffness for ≤12 coating cycles [[(HA-SS)/rhCol III]*_n_*; *n* = 3, 6, and 12] but enhanced with increased loading amount of rhCol III upon more than 12 coating cycles [[(HA-SS)/rhCol III]*_n_*; *n* = 18 and 24].

Given that [(HA-SS)/rhCol III]_3_ exhibited similar mechanical properties and biological functions to [(HA-SS)/rhCol III]_6_, as well as comparable performance between [(HA-SS)/rhCol III]_18_ and [(HA-SS)/rhCol III]_24_, as demonstrated in the results above, PLA, [(HA-SS)/rhCol III]_6_, [(HA-SS)/rhCol III]_12_, and [(HA-SS)/rhCol III]_18_ were selected for GSH treatment to further investigate their “stage-specific” property in regulating endothelial cell functions under conditions mimicking the implantation environment, using Western blotting and thrombin assays. A healthy and functional endothelium in vivo maintains an integrated and compact barrier resistant to undesirable external stimuli, which strongly dependents on tight intercellular junctions (*41*). Confluent monolayers displaying a continuous distribution of CD31 were observed in all experimental groups apart from the PLA control group before treatment with thrombin (Fig. 4F). After thrombin treatment, the HUVECs monolayer on soft [(HA-SS)/rhCol III]*_n_* (*n* = 12 and 18) coatings remained integrated, with cells tightly connected with their neighbors. Conversely, stress fibers appeared and small holes were observed within the HUVECs monolayers on PLA and [(HA-SS)/rhCol III]_6_, accompanied by the disappearance of CD31 distribution at HUVECs borders. Quantitative results indicated that the gap area was markedly higher in the PLA group than in the other groups, particularly after the addition of thrombin (Fig. 4G). Corroborating with the above results, the expression of endothelial nitric oxide synthase (eNOS) and CD31 were up-regulated by [(HA-SS)/rhCol III]*_n_* (*n* = 12 and 18) in comparison to the control PLA group, while interleukin-1β (IL-1β) and von Willebrand factor (vWF) were slightly down-regulated (Fig. 4, H to J). On a stiff substrate such as bare PLA and [(HA-SS)/rhCol III]_6_ coating in our case, the traction force sensed by HUVECs was relatively large, guiding them into a more spread phenotype. In contrast, softer substrates were governed by cell-cell interaction due to the relatively low cell-ECM interaction, thereby leading to high endothelial function, consistent with what Mauck *et al.* have unraveled that cellular behavior are regulated by the interplay between cell-cell and cell-ECM interactions (*42*–*44*).

Considering the microenvironment after stent implantation, the process of endothelial healing occurs on a soft matrix that is similar to a formulated [(HA-SS)/rhCol III]_18_. Despite the initial nondominant adhesion of endothelial cells on our soft coatings, over time, these cells can gradually achieve endothelial functionalization on dynamically softening interfaces, a marked issue that is often overlooked in current research. In summary, our soft and rhCol III-rich [(HA-SS)/rhCol III]*_n_* coatings demonstrated stage-specific property, as evidenced by mediating the adhesion of HUVECs during the initial period, followed by facilitating the high functional expression of HUVECs and the formation of healthy confluent monolayers.

### Effects of the [(HA-SS)/rhCol III]*_n_* on HUASMCs behaviors in vitro

**Fig. 5.**
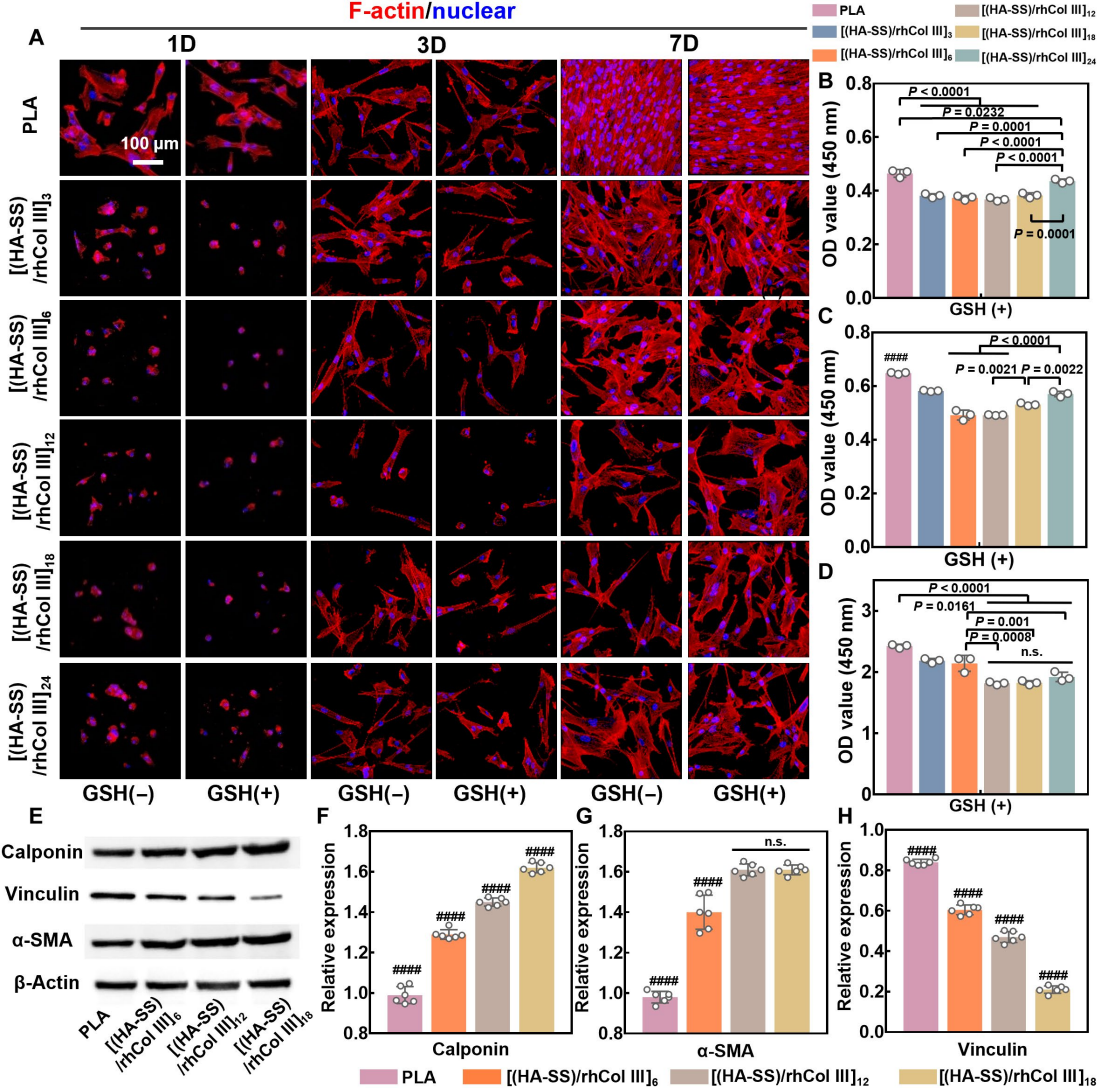
Effects of the [(HA-SS)/rhCol III]*_n_* on cell morphology and functions of HUASMCs in vitro. (**A**) Representative fluorescence images showing the adhesion and proliferation of HUASMCs were synergistically regulated by biophysical cues (stiffness) and biochemical cues (rhCol III amount). Scale bars, 100 μm. Cell viability of HUASMCs cultured on uncoated and [(HA-SS)/rhCol III]*_n_*–coated PLA sheets (*n* = 3, 6, 12, 18, and 24) with GSH treatment after (**B**) 1 day, (**C**) 3 days, and (**D**) 7 days of culture (*n* = 3 independent samples). (**E**) Typical Western blot bands, and Corresponding relative quantified data of (**F**) Calponin, (**G**) α-SMA, and (**H**) Vinculin proteins (*n* = 6 independent samples). One-way ANOVA was used for the comparisons in (B) to (D) and (F) to (H). All error bars are means ± SD (*P* values < 0.05 were considered statistically significant, and #### indicated *P* values < 0.0001 compared with other groups).

In addition to facilitating a healthy and functional endothelium, inhibiting the excessive proliferation of smooth muscle cells is also crucial for most cardiovascular implants, which is required for ideal material-host integration and avoidance of late restenosis and material failure (*26*). In healthy vessels, SMCs are contractile and unresponsive to growth signals. Nevertheless, SMCs would transit from contractile phenotype to synthetic phenotype if the vascular microenvironment pathologically changed. Upon this, we started our investigation by conducting an immunofluorescence staining assay (Fig. 5A). Within the first 3 days of culture, the adhesion and proliferation of human umbilical artery smooth muscle cells (HUASMCs) on the surfaces of the [(HA-SS)/rhCol III]*_n_* samples, with or without the addition of GSH, exhibited an initial inhibition followed by enhancement as the number of coating cycles increased. Nevertheless, after incubation was prolonged to 1 week, HUASMC proliferation did not increase with higher rhCol III loading; instead, it was substantially inhibited on the softer substrates [(HA-SS)/rhCol III]*_n_* (*n* = 12, 18, and 24) (Fig. 5, B to D and fig. S16, A to C). These data validated that the behavior of HUASMCs adhered to the [(HA-SS)/rhCol III]*_n_* coatings was initially regulated by both biophysical and biochemical cues, while substrate stiffness became the dominant regulatory factor at later stages. Integrating the results from the endothelial cell immunofluorescence assays, it became evident that the impact of the [(HA-SS)/rhCol III]*_n_* coatings on the HUASMC morphology was less pronounced compared to their effect on HUVECs. However, the proliferation behavior of HUASMCs on [(HA-SS)/rhCol III]*_n_* coatings was considerably suppressed, exhibiting a markedly lower proliferation rate compared to HUVECs at each time point (fig. S17). The above observed phenomenon might be attributed to the highly differentiated status of SMCs in an environment resembling the internal homeostatic soft matrix, which markedly limited their potential for adhesion and proliferation (*45*).

Previous studies have demonstrated that increased substrate stiffness promotes the transition of SMCs from a contractile to a synthetic phenotype, characterized by up-regulation of the synthetic marker vinculin and down-regulation of contractile markers such as α–smooth muscle actin (α-SMA) and calponin (*40**,* *46*). To further investigate the mechanisms underlying the inhibition of HUASMC adhesion and proliferation on GSH-treated substrates, we conducted Western blot analysis. As shown in Fig. 5 (E to H), the expression of α-SMA and calponin were markedly up-regulated on the softer coatings. On the contrary, there was a marked down-regulation of vinculin on stiffer coatings, indicating the enhanced focal adhesion of HUASMCs on the rigid substrates, in behavior that facilitated the transition from synthetic to the secretory phenotype of SMCs as previously reported. Summarizing the observations above, it was reasonable to conclude that soft and rhCol III-rich substrates demonstrated stage-specific property, as reflected by initially directing the adhesion of HUASMC and later promoting the conversion of HUASMC to a contractile phenotype on the coating with reduced stiffness after GSH treatment, which favored the maintenance of vascular patency following stent implantation.

From the above results, it can be concluded that the biomimetic coating with dynamic stiffness, enabling stage-specific property, favored the high functional expression of ECs and promoted the transition of SMCs to a healthy contractile phenotype. This strategy has the potential to improve the endothelial function of regenerated tissue and is expected to achieve healthy remodeling of neointima.

### [(HA-SS)/rhCol III]*_n_* modulate the immune response of macrophages

**Fig. 6.**
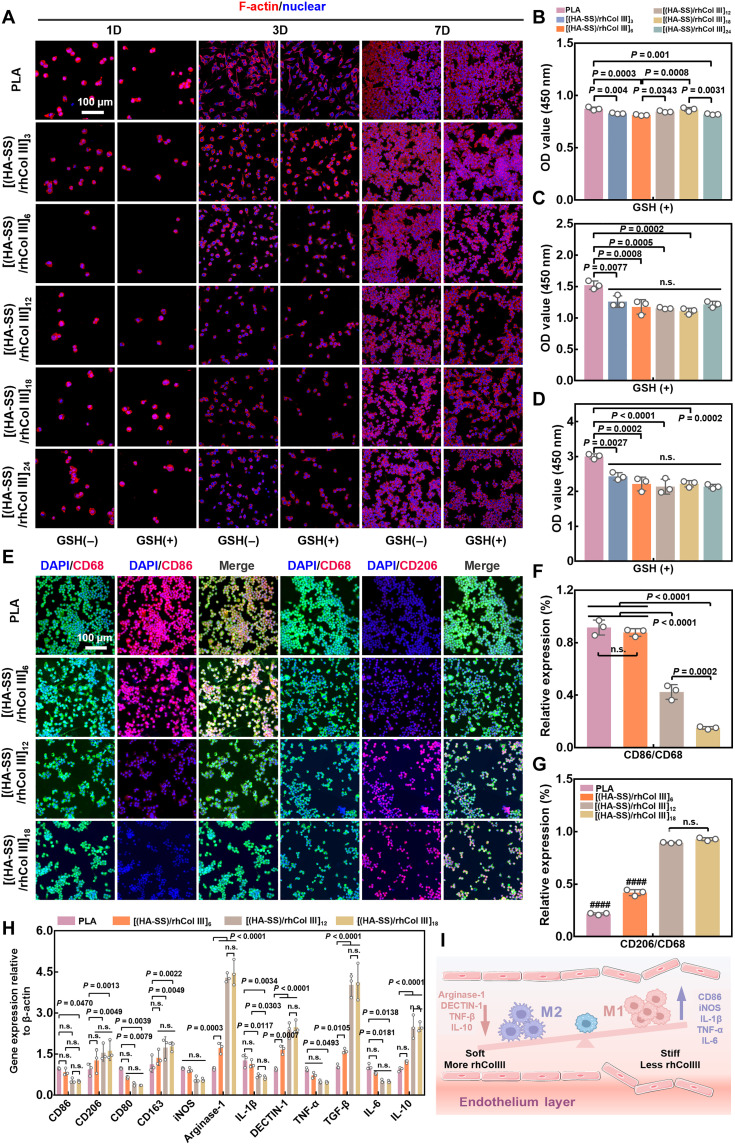
Macrophage phenotypes can be modulated by the biophysical and biochemical cues. (**A**) Representative fluorescence images stained with rhodamine (red) and DAPI (blue) of macrophages adhered to the surface of uncoated and [(HA-SS)/rhCol III]*_n_*–coated PLA sheets (*n* = 3, 6, 12, 18, and 24). Scale bar, 100 μm. Cell viability of macrophages after (**B**) 1 day, (**C**) 3 days, and (**D**) 7 days of culture (*n* = 3 independent samples). (**E**) Representative immunofluorescence images of macrophages stained for CD68 (all, green), CD86 (M1, red), and CD206 (M2, red) in different groups. Scale bar, 200 μm. Corresponding quantitative analysis of (**F**) CD86/CD68 and (**G**) CD206/CD68 in different coatings in immunofluorescence images (*n* = 3 independent samples). (**H**) Quantification of the expression of representative cytokines (i.e., pro-inflammatory cytokines CD86, CD80, iNOS, IL-1β, TNF-α, and IL-6 and anti-inflammatory cytokines CD206, CD163, Arginase-1, DECTIN-1, TNF-β, and IL-10), validated by quantitative reverse transcription polymerase chain reaction arrays. Values in the [(HA-SS)/rhCol III]*_n_* groups (*n* = 6, 12, and 18) were normalized to that in the control PLA group (*n* = 3 independent samples). (**I**) Diagram of the macrophages activated to pro-inflammatory M1 and anti-inflammatory M2 under the influence of different microenvironments. Created in BioRender. Wu, H. (2026) https://BioRender.com/syb4ne0. One-way ANOVA was used for the comparisons in (B) to (D), (F), and (G). Two-way ANOVA was used for the comparisons in (H). All error bars are means ± SD (*P* values < 0.05 were considered statistically significant, and #### indicated *P* values < 0.0001 compared with other groups).

Previous evidence suggested the relationship between macrophage morphology and polarization (*47**,* *48*). Accordingly, we first investigated the macrophage morphology regulated by our biomimetic coating in the presence or absence of GSH, and the results were shown in Fig. 6A. It should be noted that the behavior of macrophages on the [(HA-SS)/rhCol III]*_n_* surfaces during the early stages of culture was synergistically regulated by biophysical and biochemical cues as in the case of HUVEC and HUASMC, as evidenced by the fact that their adhesion and proliferation were neither merely decreased with stiffness nor increased with the loading of coating components. Obviously, as the incubation time was prolonged to 3 and 7 days, a considerable number of macrophages with the projection of abundant filopodia adhered to stiff PLA and [(HA-SS)/rhCol III]*_n_* (*n* = 3 and 6) substrates. In contrast, the cells observed on the soft [(HA-SS)/rhCol III]*_n_* (*n* = 12, 18, and 24) groups, in addition to a marked decrease in number, showed a healthy small and ovoid morphology. We also found that the number of macrophages adhered to the [(HA-SS)/rhCol III]*_n_* groups treated with GSH was further decreased in comparison to those without GSH treatment. Cell proliferation assay (Fig. 6, B to D, and fig. S18, A to C) also supported the above findings that the GSH-treated [(HA-SS)/rhCol III]*_n_* groups further inhibited macrophage proliferation compared with the untreated group. In particular, it was more pronounced at the later stage of culture, as evidenced by that all experimental [(HA-SS)/rhCol III]*_n_* groups presented marked suppression on macrophage proliferation, and there was no pronounced difference among them. The above phenomenon indicated that stiffness played a critical modulatory role in macrophage proliferation, especially at the late stage.

Our previous studies have demonstrated that rhCol III has great anti-inflammatory properties (*28*). Moreover, other previous studies have reported that substrates with high Young’s modulus would preferentially induce the polarization of macrophages toward an M1 pro-inflammatory phenotype, while the lower Young’s modulus favors the conversion to an M2 anti-inflammatory phenotype (*49*). In light of this, we are convinced about the advantages of the [(HA-SS)/rhCol III]*_n_* coatings in inflammatory regulation. Next, as in the case of HUVECs and HUASMCs, PLA and [(HA-SS)/rhCol III]*_n_* (*n* = 6, 12, and 18) were selected for GSH treatment and assessed for polarization-related phenotypes by immunofluorescence staining, with markers CD86 and CD206 characterizing M1 and M2 phenotypes, respectively. As expected, the phenotypic transition of macrophages to M1 was observed in the PLA, [(HA-SS)/rhCol III]_3_, and [(HA-SS)/rhCol III]_6_ group, as evidenced by a higher proportion of CD86^+^ cells than D206^+^ cell. In contrast, the amount of CD86^+^ cells was markedly reduced in [(HA-SS)/rhCol III]*_n_* (*n* = 12, 18, and 24) groups, with the majority of cells being CD206^+^ cells, reflecting that the cells were stimulated toward the M2 phenotype (Fig. 6, E to G). In addition, polymerase chain reaction (PCR) array also validated that the variation of macrophage phenotype was mediated by GSH-treated [(HA-SS)/rhCol III]*_n_* via the characterization of gene expression of a series of representative cytokines [i.e., pro-inflammatory cytokines CD86, CD80, inducible nitric oxide synthase (iNOS), IL-1β, tumor necrosis factor–α (TNF-α), and IL-6 and anti-inflammatory cytokines CD206, CD163, Arginase-1, DECTIN-1, TNF-β, and IL-10]. Specifically, the higher expression of Arginase-1, DECTIN-1, TNF-β, and IL-10 and the lower expression of CD80, IL-1β, and IL-6 were only observed in soft and rhCol III-rich [(HA-SS)/rhCol III]*_n_* groups (*n* = 12 and 18) (Fig. 6H), which was nicely correlated with CD86/CD206 immunofluorescence staining results.

In addition to stage-specific property exhibited in HUVECs and HUASMCs, [(HA-SS)/rhCol III]*_n_* (*n* = 12, 18, and 24) coatings with massive amounts of rhCol III and low stiffness also displayed stage-specific properties in regulating macrophages growth and function, as evidenced by the inhibition of macrophage adhesion and proliferation in the early period, followed by the facilitation of anti-inflammatory polarization of macrophages and the secretion of anti-inflammatory cytokines, providing favorable regulation of inflammation (Fig. 6I).

### Tissue compatibility of [(HA-SS)/rhCol III]*_n_* coatings in a rat model

**Fig. 7.**
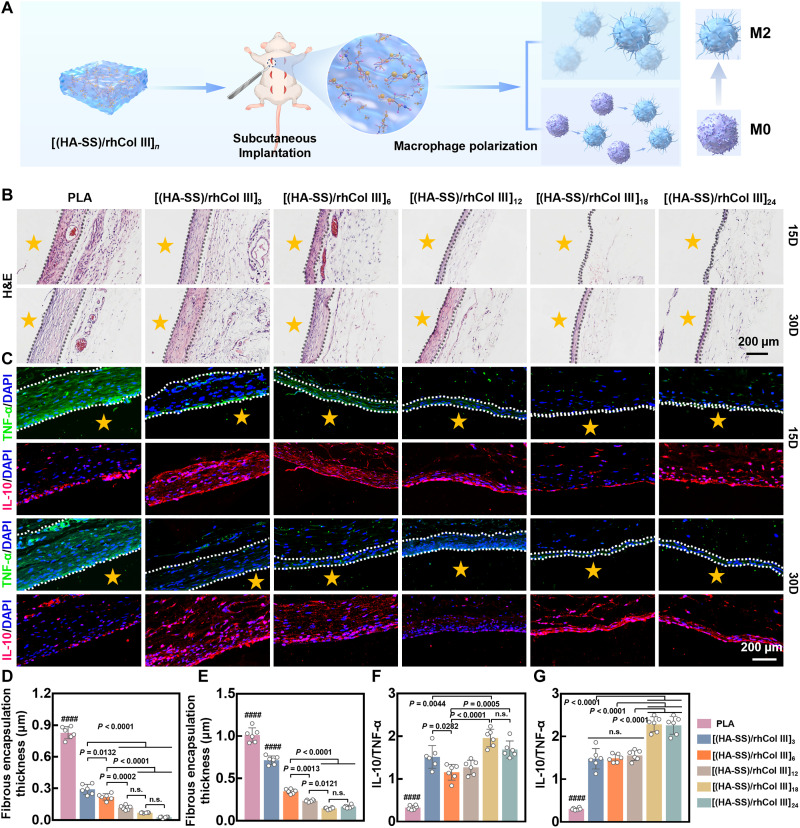
Biomimetic coatings regulate in vivo anti-inflammatory behavior. (**A**) Schematic illustrating subcutaneous implantation in the male SD rat model. Created in BioRender. Wu, H. (2026) https://BioRender.com/syb4ne0. (**B**) Representative H&E staining of uncoated and [(HA-SS)/rhCol III]*_n_*–coated PLA sheets (*n* = 3, 6, 12, 18, and 24) after 14 and 28 days of implantation. Sample placement was marked by yellow stars. Fibrous capsules were delineated in black dashed lines on H&E images. Scale bar, 200 μm. (**C**) Representative immunofluorescence images showing TNF-α (green) and IL-10 (red) expression in the fibrous capsules surrounding the various specimens. Fibrous capsules were delineated in white dashed lines on immunofluorescence images. Scale bar, 200 μm. Corresponding quantification of fibrous capsule thickness from H&E images (**D**) after 14 and (**E**) 28 days of implantation (*n* = 6 independent samples in independent animals). Corresponding quantification of IL-10/TNF-α from immunofluorescence images (**F**) after 14 and (**G**) 28 days of implantation (*n* = 6 independent samples in independent animals). One-way ANOVA was used for the comparisons in (D) to (G). All error bars are means ± SD (*P* values < 0.05 were considered statistically significant, and #### indicated *P* values < 0.0001 compared with other groups).

To further explore the in vivo inflammatory response of biomimetic coating and its potential applications, a subcutaneous implantation assay was conducted in male Sprague-Dawley rats. As the implanted samples are expected to regulate the infiltration and function of inflammatory cells, we first examined whether [(HA-SS)/rhCol III]*_n_* coatings hindered cell infiltration in vivo (Fig. 7A). Histocompatibility and inflammatory response of the materials were correlated with the thickness of the fibrous capsule surrounding the implant and the degree of inflammatory cell infiltration (*50*). Hematoxylin and eosin (H&E) staining results (Fig. 7, B to E) showed that 14 days after implantation, the substantial accumulation of inflammatory cells and thicker fibrous capsules were found in the stiff groups with less rhCol III [PLA, [(HA-SS)/rhCol III]_3_, and [(HA-SS)/rhCol III]_6_] compared to the other soft groups with a large amount of rhCol III. After 28 days, the inflammatory response remained severe in PLA, [(HA-SS)/rhCol III]_3_, and [(HA-SS)/rhCol III]_6_ groups, whereas it was markedly attenuated in the rest of the groups. The in vivo regulation of macrophage polarization with biomimetic coating was unexpectedly consistent with in vitro investigations, as demonstrated by that [(HA-SS)/rhCol III]_18_ and [(HA-SS)/rhCol III)_24_ coatings favored macrophages with more expression of the anti-inflammatory factor IL-10 and less expression of the pro-inflammatory factor TNF-α (Fig. 7, C, F, and G). Together, by adjusting the stiffness of the substrates and the content of rhCol III, we could regulate the adhesion and activation of inflammatory cells and the phenotypic transformation both in cell culture in vitro and subcutaneous model in vivo. Coatings with high Young’s modulus and less rhCol III would induce a high level of the inflammatory response, while a soft coating with more rhCol III would suppress the inflammatory response and provide a mild microenvironment for endothelial repair after stent implantation.

Among these [(HA-SS)/rhCol III]*_n_* (*n* = 3, 6, 12, 18, and 24) coatings, [(HA-SS)/rhCol III]_18_ exhibited the best overall performance, as demonstrated by properties such as comparable Young’s modulus to that of natural blood vessels, superior anticoagulation facilitated high endothelial function expression and favored conversion of smooth muscle cells to the contractile phenotype, which provided a favorable microenvironment for desirable endothelial remodeling after stent implantation. Accordingly, we selected it for the preparation of vascular stents to further investigate the safety and efficacy of the biomimetic coating in vivo.

### Stent implantation in rabbit model

**Fig. 8.**
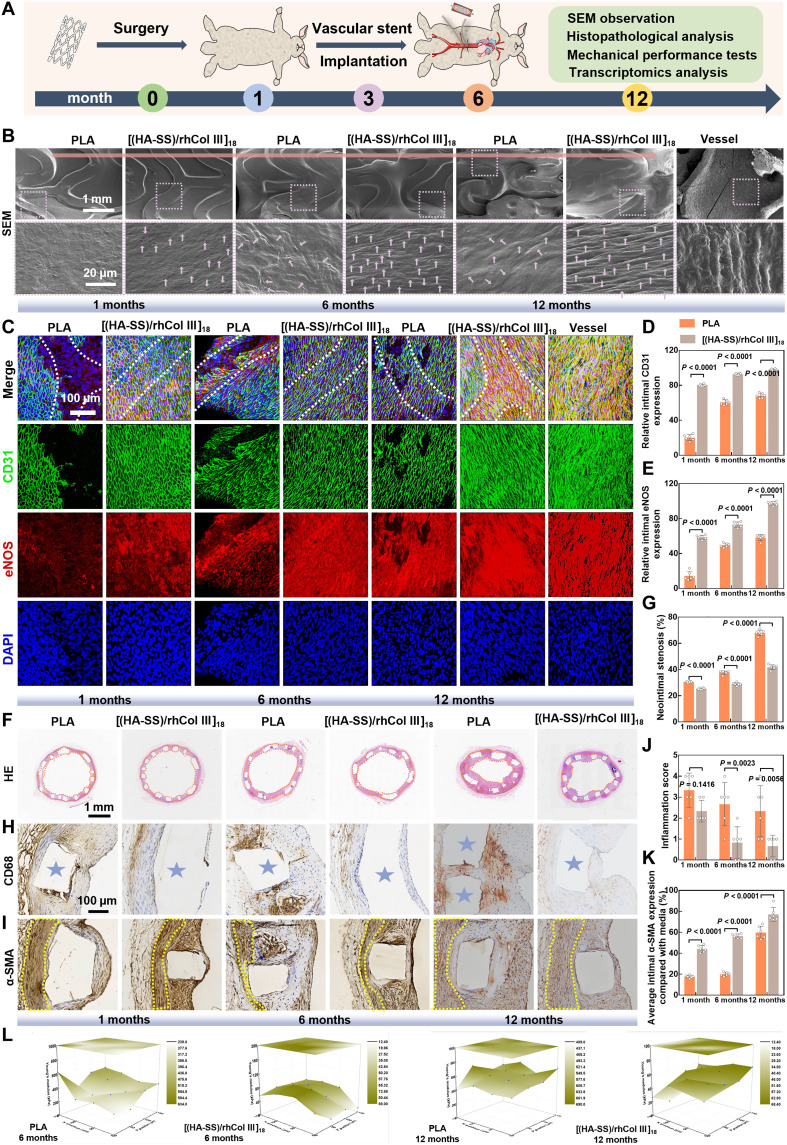
Vascular stent placement in rabbit model. (**A**) Schematic representation and detection timeline of the bare- and [(HA-SS)/rhCol III]_18_–modified stents for in vivo implantation in a rabbit model. (**B**) Representative SEM images of luminal faces. Scale bars, 1 and 20 μm. Cobblestone-like endothelial cells are indicated by purple arrows; enlarged views shown below. Three independent specimens were observed with similar results. (**C**) Representative CD31 (green) and eNOS (red) immunofluorescence images by CLSM; stent struts outlined in white dashed lines. Scale bar, 100 μm. Six independent specimens were observed with similar results. Corresponding quantification of (**D**) the relative intimal CD31 expression and (**E**) the relative intimal eNOS expression of bare PLA and [(HA-SS)/rhCol III]_18_–coated PLA stents (*n* = 6 independent specimens from independent animals). (**F**) Representative hematoxylin and eosin (HE) staining of stented arteries. Scale bar, 1 mm. (**G**) Corresponding quantification of In-stent lumen loss rate, determined from the HE images (*n* = 6 independent samples in independent animals). Representative (**H**) CD68 and (**I**) α-SMA immunofluorescence staining images of stent arteries. Scale bar, 100 μm. Purple asterisks on CD68 images marked the stent struts. The media area on SMA images was outlined with yellow dashed lines. Corresponding quantification of (**J**) the inflammation score centered around the strut and (**K**) the α-SMA expression in the intima compared with the media (*n* = 6 independent samples in independent animals). (**L**) Representative distribution of Young’s modulus of the neointima formed on the bare PLA and [(HA-SS)/rhCol III]_18_ stents after 6 and 12 months of implantation, measured by nanoindentation. Two-way ANOVA was used for the comparisons in (D), (E), (G), (J), and (K). All error bars are means ± SD (*P* values < 0.05 were considered statistically significant).

Impelled by these positive results, we proceeded to coat PLA stent with our [(HA-SS)/rhCol III]_18_ coating and tested it in animals to evaluate the biosafety and efficacy in vivo. Before that, we preliminarily examined the mechanical stability of [(HA-SS)/rhCol III]_18_–coated PLA stents by dilating them with the balloon in 37°C PBS to simulate angioplasty (fig. S19). Before balloon dilation, [(HA-SS)/rhCol III]_18_ coating uniformly covered the PLA stent with a highly consistent topography to that presented on sheets. Fortunately, no cracks or spalling were detected in [(HA-SS)/rhCol III]_18_–coated PLA stents after 1 week of circulation in PBS solution followed by balloon dilation (fig. S20, A and B). We started the exploration of the long-term reendothelialization in vivo by implanting control PLA stents and [(HA-SS)/rhCol III]_18_–coated PLA stents into the abdominal aorta of the New Zealand White rabbit (Fig. 8A). At 1 month post-stent deployment, both types of stents were completely covered by the neointima. Nevertheless, cobblestone-like cells, characteristic of endothelial cell morphology, were already observed adhering to the [(HA-SS)/rhCol III]_18_–coated PLA stents in contrast to the PLA control stents (Fig. 8B). After implantation for 3 months, the endothelial cells adherent to both two groups became denser, but the more mature phenotype in the [(HA-SS)/rhCol III]_18_–coated stents was characterized by elongated morphology and high degree of orientation (fig. S21A). Besides, the number of cells on PLA stents gradually increased with the designated time points prolonged to 6 months and to 1 year, respectively, but with loose contact with each other and poor orientation that varied markedly from that of the native intima. Encouragingly, the [(HA-SS)/rhCol III]_18_ group developed a wrinkled and furrowed morphology virtually identical to that of the native blood vessels 1 year after implantation, strongly supporting the capability of the biomimetic coating in promoting the native-like endothelium formation.

In the case of cardiovascular implants, the regeneration of a healthy and functional endothelium is extremely critical for the prevention of thrombosis and NIH as well as the restoration of proper endothelial function (*41*). To examine the degree of reendothelialization in more detail, the immunofluorescence staining was conducted. The healthy and intact endothelium is typically characterized by the expression of CD31 at the tight junctions between endothelial cells and the secretion of eNOS (*51*). Our results (Fig. 8C and fig. S20B) showed that [(HA-SS)/rhCol III]_18_–coated stents had already been covered with an intact endothelium within 1 month. In contrast, the progress of endothelialization on bare PLA stents was incomplete during this period, and even 6 months after implantation, it was still not completely covered by endothelial cells. Nevertheless, the degree of endothelialization was higher in [(HA-SS)/rhCol III]_18_–coated stents compared with bare PLA stents, as evidenced by higher CD31 and eNOS expression at any designed time points (Fig. 8, D and E, and fig. S21, C and D). Furthermore, the elongated and highly oriented morphology of the adherent endothelial cells on [(HA-SS)/rhCol III]_18_–coated stents also confirmed the formation of the more mature endothelial layer.

To explore the differences between different PLA and [(HA-SS)/rhCol III]_18_ stents concerning the capacity to suppress intimal hyperplasia, the stented arteries were harvested and then stained with hematoxylin and eosin (HE) at the designated time points. As shown in Fig. 8 (F and G) and fig. S21 (E to H), all vascular stents were completely covered, but neointimal growth rates varied markedly between groups. Overall, at all designated time points, the [(HA-SS)/rhCol III]_18_–coated stent exhibited a more pronounced inhibitory effect on neointimal formation compared to bare PLA stents (see the Supplementary Materials for detailed discussion).

On the basis of the presented great anti-inflammatory and suppression of smooth muscle hyperproliferation by our [(HA-SS)/rhCol III]_18_ coating in vitro, we further evaluated its effectiveness in vivo by histological analyses on the cross sections of the stented arteries. The results (Fig. 8, H and J, and fig. S20, I and K) demonstrated that bare PLA stents induced moderate to severe inflammation within 3 months, with a slight reduction in inflammation observed after 6 months. In sharp contrast, the [(HA-SS)/rhCol III]_18_–coated stents induced only minimal inflammation during the implantation period. In addition to inflammation, restenosis is also related to the SMC phenotype. Contractile SMC with low proliferative and migratory capacity is important in maintaining vascular elasticity and contraction, which is the basis for inhibiting excessive NIH (*52*). In contrast to the expression of CD68, the α-SMA expression level of [(HA-SS)/rhCol III]_18_ was markedly higher than that of bare PLA stents at any designated time point (Fig. 8, I and K, and fig. S20, J and L). To more comprehensively evaluate the effect of our [(HA-SS)/rhCol III]_18_ coating on the restoration of the endothelium, in addition to the above histopathological analysis, we also characterized the neonatal intima surrounding the stents in the biophysical aspect by measuring Young’s modulus. At 6 and 12 months post-stent deployment, Young’s modulus, collected by the nano-indentation equipment, was uniformly distributed in the [(HA-SS)/rhCol III]_18_ group with an average value in the range of 30 to 50 kPa, which was similar to that of the healthy intima layer. However, the values of Young’s modulus were markedly increased to 200 and 600 kPa for the control PLA group at the same time points, respectively, which was even much higher than that of the atherosclerotic arteries and was negative for the ideal intimal repair (Fig. 8L). Combining all the results analysized above, it is reasonable to believe that [(HA-SS)/rhCol III]_18_–coated stents are more favorable for the restoration of healthy native endothelium in comparison with bare PLA stents.

Furthermore, we performed a literature review and compared key histological parameters—thrombogenicity, endothelialization, and neointimal thickness—across various clinically approved and investigational stents, including first- and second-generation DESs, BRSs, and functional platforms, to better demonstrate the advantages of our [(HA-SS)/rhCol III]_18_–coated stent (table S2) (*3**,* *43**,* *53**–**77*). Data were derived from large-animal pathologic models or human patient studies. Specifically, conventional DESs effectively suppress NIH but are frequently associated with delayed endothelialization. BRSs, in turn, facilitate endothelial repair yet tend to induce thicker neointimal formation. Other functional stents commonly exhibit high thrombogenicity or limited efficacy in NIH suppression. In contrast, our [(HA-SS)/rhCol III]_18_–coated stent showed very low thrombogenicity, complete endothelial regeneration within 1 month, and superior NIH suppression. Collectively, these results indicated that the [(HA-SS)/rhCol III]_18_ stent performed comparably to or better than existing technologies in several critical aspects, supporting its potential for marked improvement and clinical translation.

### Transcriptome analysis of stented aorta revealed vascular neointimal remodeling mechanism

**Fig. 9.**
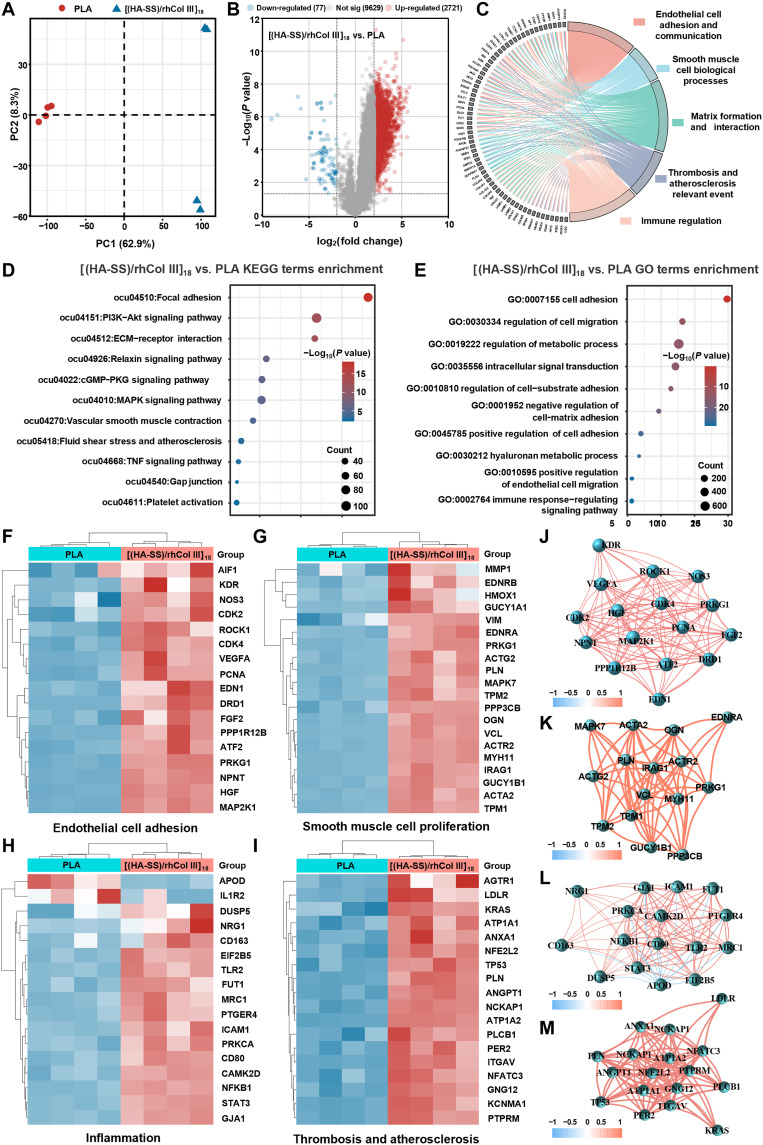
Transcriptome analysis illustrating [(HA-SS)/rhCol III]_18_–coated stents favored the restoration of native vascular endothelium. (**A**) PCA of different groups. (**B**) Volcano plots of DEGs in the PLA group versus the [(HA-SS)/rhCol III]_18_ group, which were screened out under the settings of thresholds of │log_2_FC│ > 2 and *P* < 0.05. Down-regulated and up-regulated genes were colored in blue and red, respectively. (**C**) Circulation visualization results of pathway-gene enrichment analysis. (**D**) Regulated pathway in enriched KEGG terms of [(HA-SS)/rhCol III]_18_ versus PLA analysis. (**E**) Regulated biological processes in enriched GO analysis of [(HA-SS)/rhCol III]_18_ group and PLA group. Heatmap analysis and corresponding String interaction network of unique DEGs involved in (**F** and **J**) endothelial cell adhesion, (**G** and **K**) smooth muscle cell proliferation, (**H** and **L**) inflammation, and (**I** and **M**) thrombosis and atherosclerosis analysis. Two-sided Student’s *t* test with multiple testing corrections was used in (B), (D), and (E).

To uncover why [(HA-SS)/rhCol III]_18_–coated PLA stents favored desirable native endothelium restoration, we collected stented aortas 3 weeks postimplantation and performed transcriptomic analyses. Principal components analysis (PCA) was performed and results showed high biological replicability among the four independent specimens in each group, with transcriptome data available for further analysis (Fig. 9A). Next, we screened out differentially expressed genes (DEGs) under the threshold treatments of │log_2_ fold change (FC)│ > 2 and *P* < 0.05. According to the volcano plot results, 2721 considerably up-regulated and 77 considerably down-regulated genes were detected in the [(HA-SS)/rhCol III]_18_ group compared to the PLA control group, respectively (Fig. 9B). To better understand the impact of altered genomic expression profiles on the vascular endothelial restoration and remodeling, the above-mentioned DEGs were analyzed by enriched Kyoto Encyclopedia of Genes and Genomes (KEGG) pathways and enriched Gene Ontology (GO) terms. The relevant enriched KEGG pathways of [(HA-SS)/rhCol III]_18_ versus PLA and its corresponding circular visualization analysis were illustrated in Fig. 9 (C and D), which could be classified into five components: (i) regulation of endothelial cell adhesion and communication: phosphatidylinositol 3-kinase–Akt signaling pathway; (ii) regulation of smooth muscle cell biological processes: cyclic guanosine 3′,5′-monophosphate (cGMP)–cGMP-dependent protein kinase (PKG) signaling pathway, vascular smooth muscle contraction, mitogen-activated protein kinase signaling pathway, and relaxation signaling pathway; (iii) immune regulation: TNF signaling pathway; (iv) thrombosis and atherosclerosis relevant event: platelet activation, lipid and atherosclerosis, and fluid shear stress and atherosclerosis; (v) ECM interactions: focal adhesion, gap junction, and ECM-receptor interaction. The GO database analysis results revealed that the regulated DEGs in [(HA-SS)/rhCol III]_18_ versus PLA were primarily enriched in the regulation of cell migration and adhesion, regulation of the metabolic process, positive regulation of endothelial cell migration, immune response regulating signaling pathway, and regulation of cell-matrix adhesion and cell-substrate adhesion (Fig. 9E).

A specific heatmap of DEGs and their corresponding protein-protein interaction networks screened from the relevant GO and KEGG analysis involved four typical categories: endothelial cell adhesion, smooth muscle cell proliferation, inflammation, and thrombosis and atherosclerosis, as indicated by the results in Fig. 9 (F to M). Encouragingly, [(HA-SS)/rhCol III]_18_ treatment contributed to the rapid endothelium restoration of damaged vascular tissue after stent implantation since some genes facilitating endothelial cell proliferation such as *AIF1*, *ATF2*, *HGF*, *PPP1R12B*, and *ROCK1* were exclusively up-regulated compared to the control PLA group. Moreover, the [(HA-SS)/rhCol III]_18_ favored the maintenance of vascular homeostasis and health, as evidenced by an impressive up-regulation of genes positively associated with endothelial cell function (Fig. 9, F and J). Compared with the PLA control group, we observed a marked and unique up-regulation of the synthetic phenotypic SMC markers (*ACTA2*, *MYH11*, *MMP1*, *VIM*, *VCL*, *TMP1*, *TMP2*, and *OGN*) in the [(HA-SS)/rhCol III]_18_ treatment, which facilitated the inhibition of excessive smooth muscle cells proliferation. Besides, these genes associated with vascular relaxation [*PPP3CB* (*78*), *PRKG1* (*79*), and *HMOX1* (*43*)] and the production of NO molecules (*GUCY1A1* and *GUCY1B1*) were also remarkably up-regulated in [(HA-SS)/rhCol III]_18_ group, which are reportedly to induce the relaxation of smooth muscle cells and assist in vasodilatation of blood vessel, thereby preventing the occlusion of stented artery (Fig. 9, G and K). Inflammation-related gene heatmap results revealed that the [(HA-SS)/rhCol III]_18_ treatment favored a positive regulation of the immune response, as evidenced by the notable up-regulation of *PTGER4*, *FUT1*, *CD80*, *NFKB1*, *STAT3*, and *GJA1* and down-regulation of *APOD*. In addition, [(HA-SS)/rhCol III]_18_ induced the up-regulation of the M2 macrophage markers (*CAMK2D*, *MRC1*, and *CD163*) and the anti-inflammatory genes [*DUSP5*, *NRG1* (*43*), *TLR2*, and *PRKCA*] concerning PLA controls group, which could effectively suppress the inflammatory response and thereby provided a favorable local microenvironment for native vascular endothelial restoration (Fig. 9, H and L). The control PLA group also favored LST and atherosclerosis, as indicated by the up-regulation of *AGTR1*, *ANXA1*, and *NFATC3*. In stark contrast, a marked up-regulation of genes that positively contribute to vascular remodeling (*ANGPT1* and *NFE2L2*) and cancer suppressor genes (*TP53*, *PER2*, *PTPRM*, *GNG12*, and *PLCB1*) was detected in the [(HA-SS)/rhCol III]_18_ group (Fig. 9, I and M).

Summarizing the positive transcriptome analysis results above, it can be concluded that the PLA stents induced neointimal dysfunction, thus potentially stimulating follow-up LST and ISR. However, the neointima around the [(HA-SS)/rhCol III]_18_–coated stents sustained healthy endothelial cell function, favored the vascular patency by regulating the transition of SMCs to contractile phenotype, and suppressed the inflammatory response by mediating the transition of macrophages to the M2 phenotype. Overall, the collaboration of the above-mentioned functions supported the ideal restoration of native vascular endothelium.

## DISCUSSION

In conclusion, a biomimetic coating formulation composed of HA grafted with disulfide bonds and meticulously tailored recombinant humanized collagen type III (rhCol III) was developed, which not only suppressed adverse interfacial responses in the early stages post-stent implantation but also altered and modulated cell behaviors and functions at the later stages under cardiovascular system stimuli (GSH and H_2_O_2_), providing a “stage-specific properties” platform for desired neointimal remodeling and functional endothelialization. In vitro QCM-D assay and Young’s modulus test confirmed that the stiffness of the [(HA-SS)/rhCol III]*_n_* coatings would be dynamically reduced approaching that of native blood vessels with GSH and H_2_O_2_ treatment. The effectiveness of [(HA-SS)/rhCol III]*_n_* in markedly suppressing the nonspecific adsorption and denaturation of plasma proteins and preventing thrombi formation was verified by in vitro and in vivo hemocompatibility assays. Furthermore, it demonstrated stage-specific properties that initially suppressed adverse interfacial responses and directed cell adhesion, followed by facilitating the formation of confluent endothelial cell monolayers with high functional expression, the conversion of smooth muscle cells to contractile phenotype, and the anti-inflammatory polarization of macrophages. As a result of these synergistic effects, our [(HA-SS)/rhCol III]_18_–coated stents displayed desirable restoration of the endothelium 12 months after implantation in the abdominal aorta of rabbits, as evidenced by a Young’s modulus comparable to that of native vessels and the native-like endothelium function.

Note that this study still has certain limitations in terms of anticoagulant selection and animal experimental design. Although the anticoagulant performance of the coating has been consistently verified in vitro and in vivo, sodium citrate will be used as the standard anticoagulant for platelet preparation in future studies to further improve the clinical relevance. In addition, the small-animal models used in this study for coronary stents might not be good enough to accurately reflect the real clinical application scenarios. Furthermore, our findings were conducted on healthy animals, whereas the biophysical and biochemical functions of our [(HA-SS)/rhCol III]_18_ coating on pathological models might not be exactly the same. A thorough investigation in a follow-up study on whether [(HA-SS)/rhCol III]_18_ coating remains effective in promoting the ideal regeneration of the endothelium in a large-animal model of porcine and also in a small-animal pathological cardiovascular model is warranted to ensure its safety and efficacy for future clinical applications.

Nevertheless, these limitations are negligible in light of the value in potential applications of our [(HA-SS)/rhCol III]_18_ coating. As a strategy of “healthy neointima healing,” the [(HA-SS)/rhCol III]_18_ coating shows the potential for ideal vascular tissue healing in terms of Young’s modulus and endothelial functions of native-like vascular vessels, something that cannot be realized with current stent products. Moreover, the biomimetic strategy with dynamic stiffness enabling stage-specific properties provided a promising biomimetic approach to armor vascular implants for enhanced vascular tissue healing.

## MATERIALS AND METHODS

### Materials and reagents

PLA sheets and stents were provided by Sichuan Xingtai Pule Medical Technology Co. Ltd. (Chengdu, China). Dopamine hydrochloride (Dopa), PEI [weight-average molecular weight (*M*_w_) = 25,000], HA (HA, *M*_w_ = 100,000), cystamine dihydrochloride (Cys), and 1, 6-diaminohexane dihydrochloride were purchased from Adamas Reagent Co. Ltd. (Shanghai, China). 2-(*N*-morpholino) MES, EDC, NHS, and GSH were purchased from Sigma-Aldrich. Recombinant peptides derived from human type III collagen (named the tailored rhCol III in this work, average *M*_w_ ∼ 45 kDa) were meticulously tailored based on our requirements using advanced structural biology and genetic engineering technologies. Specifically, rhCol III was synthesized by 16 tandem repeats of the T16WTp (sequence: Ac-GERGAPGFRGPAGPNGIPGEKGPAGERGAP-NH_2_). The biological reagents for evaluating blood and cell compatibility were purchased from professional manufacturers and mentioned in the experimental section.

### Synthesis and characterization of HA-SS

The preparation of HA-SS was based on the established method (*25*). Briefly, the EDC (287.6 mg) and NHS (202.7 mg) were dissolved in 20 ml of HA solution (200 mg) for 2 hours to activate the carboxyl groups. After that, Cys (337.8 mg) was added, and then the pH of the solution was adjusted to 7.0 with stirring for 48 hours to facilitate the grafting of Cys to HA. Then, the mixture was fully dialyzed with 4000 translucent molecular weights dialysis bag for 3 days and freeze-dried with the products, denoted as HA-SS. Note that the fabrication procedures for HA-CC (hyaluronan acid-grafted 1, 6-diaminohexane dihydrochloride) were identical to those described in HA-SS. ^1^H NMR spectra were used to examine HA-SS and HA-CC.

### Fabrication of [(HA-SS)/rhCol III]*_n_*

Preparation of [(HA-SS)/rhCol III]*_n_* multilayer films was as follows: The PLA substrates, including sheets and stents, were first pretreated with Dopa and PEI to fabricate a classic mussel-mimicking amino-amplified surface (*26*). Briefly, the substrates were immersed in Dopa solution [0.2 mg/ml in 10 mM tris buffer (pH 8.5)] for 45 min, sequentially an equal volume of PEI solution [40 mg/ml in 10 mM tris buffer (pH 8.5)] was added to the above reaction solution for another 30 min. The substrates coated PEI-PDA coating was obtained after rinsing with deionized water three times.

The PEI-PDA–modified PLA substrates were sequentially dipped into HA-SS (2 mg/ml; the pH was adjusted to 5.4 using 10 M NaOH solution) and rhCol III (1 mg/ml; the pH was adjusted to 7.4 using 10 M NaOH solution) solutions for 15 and 20 min, respectively, as a coating cycle. Following the *n* coating cycles (*n* = 3, 6, 12, 18, and 24), samples were immediately immersed into the MES buffer (0.05 M, pH 5.40) containing EDC (0.05 M) and NHS (0.05 M) overnight at 4°C to obtain more stable stiffness-regulated multilayer films, which were referred to as [(HA-SS)/rhCol III]*_n_*. Notably, the fabrication procedures for ((HA-CC)/rhCol III)*_n_* were the same as those described in [(HA-SS)/rhCol III]*_n_*. Last, the samples were stored in a 4°C refrigerator for the following physiochemical characterization, cell culture, and stent implantation.

### Physiochemical characterization of [(HA-SS)/rhCol III]*_n_*

The assembly of the [(HA-SS)/rhCol III]*_n_* coatings prepared with FITC-labeled Col III was observed by a confocal laser scanning microscope (Leica SP5, Germany). The amount of disulfide bonds before and after [(HA-SS)/rhCol III]*_n_* modification was quantified by a free sulfhydryl assay kit with 5,5′-dithiobis-(2-nitrobenzoicacid) (DTNB; Beyotime Biotechnology, China). The micromorphology of the [(HA-SS)/rhCol III]*_n_* coatings was investigated by scanning electron microscope (SEM, FEI Nova Nano 450). WCAs were evaluated using Attention Theta (Biolin Scientific, Sweden). The chemical bond of coatings was identified by XPS (Thermo Scientific ESCALAB 250Xi, USA) and FTIR spectra (ATR-FTIR, Spectrum One, Nicolet).

The real-time monitoring of the [(HA-SS)/rhCol III]*_n_* coating construction process was performed with the QCM-D (QSense Analyzer, Biolin, Sweden). Briefly, mussel-mimicking amino-amplified PDA-PEI coatings were prepared on 5-MHz gold-plated quartz crystal before testing. Then, the reaction solution with the identical concentration as described in the coating construction procedure was passed into the chamber at a flow rate of 50 μl/min. To verify the dynamic hardness properties of our biomimetic coating, the following steps were conducted: (i) the [(HA-SS)/rhCol III]_12_ and ((HA-CC)/rhCol III)_12_ coatings were prepared on quartz crystals following the procedure mentioned in coating fabrication; and (ii) 10 mM GSH and 1 mM H_2_O_2_ were passed into the reaction chamber at a flow rate of 50 μl/min. The data analysis was conducted with Dfind Smartfit modeling in QSense Dfind software.

### AFM-based test for local stiffness measurement

The stiffness of the bare, PDA-PEI-, [(HA-SS)/rhCol III]*_n_*, and ((HA-CC)/rhCol III)*_n_*–modified (*n* = 3, 6, 12, 18, and 24) PLA sheets was observed by AFM (Bruker). Briefly, the [(HA-SS)/rhCol III]*_n_*–coated PLA were placed in PBS, 10 mM GSH, and 1 mM H_2_O_2_ for 1 day and then measured by AFM (Bruker) using tapping mode with a probe (0.06 N/m; SNL-10, Bruker). Force-displacement curve was recorded during the whole process from which the local Young’s modulus could be calculated.

### Hemocompatibility evaluation

All animal experimental procedures were kept to a strict protocol approved by the Sichuan Provincial Laboratory Animal Ethics Committee and performed following the Guidelines for the Care and Use of Laboratory Animals of Sichuan University (approval no. KS2020394). Experiments involving human blood were approved by the Institutional Ethics Committee of Sichuan University, and informed consent was obtained from all volunteers. The male New Zealand white rabbits (weighing 2.5 to 3.0 kg) used in the study were supplied by the Chengdu Dossy Experimental Animals Co. Ltd.

Uncoated and [(HA-SS)/rhCol III]*_n_*–coated PLA (*n* = 3, 6, 12, 18, and 24) sheets were coincubated with FITC-hFg (Dalian Meilun Biotechnology Co. Ltd., China) at 37°C for 4 hours, 1 day, and 3 days, respectively. Then, control bare PLA and [(HA-SS)/rhCol III]*_n_*–coated PLA were washed with NaCl solution, and the fibrinogen adsorption behavior on the surface of the samples was investigated by confocal laser scanning microscope (CLSM) imaging. Another set of the above samples were incubated with diluted plasma specimens for 2 hours at 37°C, followed by the analysis of conformational changes of adsorbed fibrinogen by Fg-γ ELISA kits (COIBO BIO, China), as detailed in the instruction manual.

Fresh PRP was collected from healthy human volunteers, prepared by centrifugation and stored in medical-grade vacuum blood collection tubes containing EDTA as the anticoagulant. Samples were incubated with fresh PRP at 37°C for 2 hours, then removed, and fixed with 4% paraformaldehyde overnight. Platelet adhesion and activation on sample surfaces were observed by SEM.

New Zealand white rabbits were used for ex vivo AV shunt assay according to the previous reports (*28*). In short, the [(HA-SS)/rhCol III]*_n_* coatings (*n* = 3, 6, 12, 18, and 24) were prepared on the PVC tubes as mentioned in the coating construction procedure, and then the rabbits were anesthetized by intravenous injection of pentobarbital sodium salt solution [saline (25 mg ml^−1^), 0.8 ml kg^−1^] through the ear margin. After that, the left carotid artery and right jugular vein of the New Zealand white rabbits (weighing 2.5 to 3.0 kg) were carefully isolated and connected with the modified tubes to form the AV extracorporeal circuit. After 2-hour circulation, the circuit was removed and rinsed with NaCl solution thoroughly and then photographed. The thrombogenicity of the samples was evaluated by measuring the occlusion and patency of the modified tubes and the weight of the thrombus formed on the surface of the samples. The occlusion of the ((HA-SS)/rhColIII)*_n_*–coated PVC tubes was calculated by ImageJ software. The micromorphology of the modified PVC tubes was observed by SEM after dehydration.

In vivo stent implantation was conducted to further evaluate the thromboprotective effect of the ((HA-SS)/rhColIII)*_n_* coatings (*n* = 6, 12, and 18) postimplantation. Briefly, rabbits were anesthetized as described above, and then the femoral artery was carefully isolated for 3 cm following arterial penetration. Afterward, the uncoated and [(HA-SS)/rhCol III]*_n_*–coated PLA vascular stents (Φ2.75 mm by 13 mm) were implanted into the abdominal aorta of the rabbits using a balloon under 8-atm pressure for 30 s. After 3 weeks of implantation, the arteries with implanted stents were collected, washed, fixed with 4% glutaraldehyde, and lastly cut along the long axis for SEM analysis.

### Cell culture

Primary human umbilical vein endothelial cells (HUVECs, catalog no. STCC12103) were purchased from Service-bio Technology Co. (China). Primary HUASMCs (catalog no. C-12500) were obtained from Procell Life Science&Technology Co. Ltd. (USA). Mouse mononuclear macrophage cells (RAW 264.7, catalog no. C7505) were purchased from Beyotime Biotechnology (China). HUVECs and RAW 264.7 were cultured with RPMI 1640 (catalog no. 31870082, Gibco) media supplemented with 10% (v/v) fetal bovine serum (FBS; catalog no. A3160901, Gibco) and 1% (v/v) penicillin-streptomycin (P/S) (catalog no. 15640055, Gibco). HUASMCs were cultured in smooth muscle cell medium (catalog no. 1101, ScienCell Research Laboratories) containing 2% (v/v) FBS (catalog no. 0010), 1% (v/v) smooth muscle cell growth factor (smooth muscle cell growth supplement, catalog no. 1152), and 1% (v/v) P/S (catalog no. 0503). The cells used in this study were incubated in incubators at 37°C with 5% CO_2_. Cells were treated with trypsin (0.05%, w/v)/EDTA (0.53 mM) solution once ∼80% confluence was accomplished and centrifuged for collection of cell pellet, followed by resuspension in their growth medium for subculturing or subsequent cytocompatibility assays.

### Cell proliferation and morphology assay

The bare and [(HA-SS)/rhCol III]*_n_*–coated PLA (*n* = 3, 6, 12, 18, and 24) sheets (1 cm by 1 cm) were placed in a 24-well cell culture plate and sterilized with ethylene oxide at 37°C for 12 hours. Next, the cell suspensions (1 ml) with a density of 2 × 10^4^ cells/ml were added to the substrates and incubated for 1, 3, and 7 days, respectively. Notably, two groups were established in vitro cell experiments: one group without the addition of GSH and another group with the addition of 10 μM GSH. At the designated time, the cells were gently cleaned thrice with PBS, and then fluorescence staining was conducted with rhodamine-conjugated phalloidin (Phalloidin-TRITC) and 4′,6-diamidino-2-phenylindole (DAPI) dyes following the protocol provided by the manufacturer. Afterward, the morphology of cells adhered to the samples was photographed using CLSM. Moreover, the proliferation of cells was detected using Cell Counting Kit-8 (Dojindo Molecular Technologies, Japan).

### Integrity of HUVECs monolayer

The suspension of HUVECs with a density of 2 × 10^4^ cells/cm^2^ was seeded onto the specimens. The medium supplemented with GSH was changed every 2 days until the cells reached a tightly connected monolayer. To assess the integrity of HUVECs monolayers, thrombin (2 U/ml; T6884, Sigma-Aldrich) was added to the specimens with confluent monolayers and processed for 15 min. Afterward, they were treated with the following reagents: 4% formaldehyde solution for 20 min, PBS containing 0.5% Triton X-100 solution for 1 hour, PBS containing 5% bovine serum albumin (BSA) solution for 12 hours, Rabbit anti-human CD31 (PECAM-1) monoclonal antibody for 6 hours, phalloidin-TRITC (1 μg ml^−1^ in PBS), and DAPI (5 μg ml^−1^ in PBS) for 6 hours. After extensive washing with PBS, the cells on the substrates were photographed using a whole slide scanner (VS200, Olympus).

### Western blotting analysis

The total proteins of the cells were extracted with radioimmunoprecipitation assay lysis buffer once they reached about 80% confluence and quantitatively determined by bicinchoninic acid protein assay kit (Thermo Fisher Scientific, Waltham, MA, USA) Switzerland). After that, the protein was separated with 10% SDS–polyacrylamide gel electrophoresis gels and transferred to polyvinylidene difluoride membranes (0.45 mm; Solarbio, Beijing, China). Next, the membranes were blocked with tris-buffered saline with Tween 20 (TBST) containing 5% BSA (Amresco) for 1 hour and then incubated with certain primary antibodies at 4°C overnight. After being taken out and rewarmed for 30 min the next day, the membranes were washed gently with TBST solution and then incubated with the secondary antibodies for 1 hour at room temperature without light. The bands were colored by electrochemiluminescence (ECL) reagents (Thermo Fisher Scientific) and the images were collected with Omega Lum G (Aplegen, USA). Identified primary antibodies used for assessing the function of HUVECs included IL-1β, vWF, CD31, and eNOS; those indicated for HUASMCs included calponin, α-SMA, and vinculin. The corresponding secondary antibodies included Alex Fluor 488 goat anti-mouse immunoglobulin G (IgG) (1:500; catalog no. A-11001, Invitrogen, USA), and Alexa Fluor 647 donkey anti-rabbit IgG (1:500; catalog no. A-31573, Invitrogen, USA).

### Reverse transcription PCR array

After 3 days of cultivation, RNA was extracted from the cells grown on different specimen surfaces using TRIzol reagent (Thermo Fisher Scientific, USA), followed by thorough rinsing with ribonuclease-free water (Thermo Fisher Scientific, USA). The RNA quality, in terms of concentration and purity, was confirmed to meet the standards, and then the RNA was reverse-transcribed into cDNA using iScript cDNA Synthesis Kit (Bio-Rad, USA), which served as a template for the subsequent polymerase chain reaction (PCR) amplification. Following this, gene expression levels were analyzed on a CFX96 instrument (Bio-Rad) with the addition of AceQ qPCR SYBR Green Master Mix (Vazyme) and specific primers. Line Gene 9600 Plus Software (FQD-96A, Bio-Rad, Japan) was used to quantify the relative gene expression, applying the 2^-ΔΔ*Ct*^ formula. To characterize the distinct phenotypes of macrophages, we quantified both pro-inflammatory markers such as CD86, CD80, iNOS, IL-1β, TNF-α, and IL-6, as well as anti-inflammatory cytokines CD206, CD163, Arginase-1, DECTIN-1, TNF-β, and IL-10. For a comprehensive list of primers associated with macrophage polarization, refer to table S1.

### In vivo subcutaneous implantation studies

Twelve Male Sprague Dawley (SD) rats weighing 250 to 300 g were anesthetized with pentobarbital (25 mg/ml) at a dose of 0.8 × 10 ml/kg. For in vivo subcutaneous implantation experiments, six parallel samples were set up for each sample at each time point. In short, the dorsal skin of rats was separated carefully from the subcutaneous muscles using hemostatic forceps, followed by the implantation of bare and [(HA-SS)/rhCol III]*_n_*–coated PLA sheets (*n* = 3, 6, 12, 18, and 24, 0.8 cm by 0.8 cm) into both sides of the subcutaneous pockets (three pockets on the back of each rat). After 2 and 4 weeks of implantation, the capsules wrapped bare, modified PLA sheets were explanted, and then SD rats were euthanized with an overdose of pentobarbital solution. Following 48-hour fixation of the capsule tissues with 4% paraformaldehyde, H&E staining and immunofluorescence staining (TNF-α and IL-10) were performed to assess the inflammatory response of [(HA-SS)/rhCol III]*_n_* coatings.

### Mechanical stability tests

The [(HA-SS)/rhCol III]_18_ coatings were prepared on PLA stents as mentioned above, which were then press-gripped. Subsequently, the [(HA-SS)/rhCol III]_18_–modified stents were dilated in PBS solution at 37°C for 40 s at 8 atm. After that, the stents were gently placed in commercially bare tubes, which were in a closed circuit with the peristaltic pump, and were processed at 37°C in a flowing system containing PBS with a flow rate of 50 μl/min. After circulating for 1 week, the samples were removed. The surface morphology of different samples was investigated by SEM.

### Stent implantation in rabbit model

The [(HA-SS)/rhCol III]_18_–modified PLA stents were implanted into the abdominal aortic vessels of the healthy New Zealand white adult rabbits (weighing 2.5 to 3 kg) to investigate the effects on the reendothelialization and antirestenosis activity compared with bare PLA stents (assumed as control). Before the procedure, bare and [(HA-SS)/rhCol III]_18_–coated PLA stents were sterilized with ethylene oxide for 12 hours at 37°C. The rabbits were anesthetized with pentobarbital sodium (25 mg/ml, 0.7 ml/kg), and then the femoral artery was isolated for an adequate length (3 cm) for subsequent arterial puncture. Afterward, the stent was delivered from the femoral artery to the abdominal aorta and dilated using a balloon under 8-atm pressure for 40 s. Following the withdrawal of the catheter and the ligation of the artery, the rabbits were administered ampicillin sodium salt solution with intramuscular injection (10 mg/ml, 1 ml/kg). In addition, aspirin (5 mg/kg) and clopidogrel (3 mg/kg) were administered for three continuous days after stent implantation. After 1, 3, 6, and 12 months of implantation, the stented aorta tissues were harvested, and then the rabbits were euthanized with an overdose of pentobarbital solution. After being fixed with 4% formaldehyde for 2 days, the collected stented aortas tissues were equally divided into three segments immunofluorescence staining, immunohistochemistry staining, and SEM observations, respectively.

### Histopathological analysis

Tissues fixed with 4% paraformaldehyde were taken out, including the capsules and stented aorta tissues, followed by dehydrated and paraffin-embedded treatments successively. Then, slices with 4-μm thickness were cut from the proximal, middle, and distal ends of the tissues individually for immunostaining using a hard tissue microtome (HM 340E, Thermo Fisher Scientific, USA).

For the obtained slices of capsule tissues, one portion was stained with H&E following the protocol provided by the vendor, and another portion was processed for immunofluorescence staining with the following treatment: Sections were dewaxed using xylene for 5 min and then blocked with 3% H_2_O_2_ for 15 min. Subsequently, they were incubated with the specific primary antibodies such as CD206 and CD86 overnight at 4°C, and then with the corresponding secondary antibodies for 10 min at 37°C. After that, they were color-developed using Opal 570 reagent for 10 min and then stained with DAPI dyes for 5 min.

The prepared slices of stented aorta tissues were treated with H&E staining and immunohistochemical staining (CD68 and α-SMA). Furthermore, the remaining stented aorta segments were processed for CD31 and eNOS immunofluorescence staining. Briefly, they were permeabilized and blocked with goat serum solution containing 0.3% Triton X-100 for 20 min and then incubated with a primary antibody mixture at 4°C overnight. After that, they were incubated with the secondary antibodies for 1 hour at room temperature, and nuclear were stained by DAPI dyes for 5 min. Last, the slides were imaged with a whole slide scanner (VS200, Olympus), and the tissue samples were photographed using CLSM. In-stent lumen loss rate was calculated from directly measured the slices based on H&E images using ImageJ software, e.g., in-stent lumen loss rate: (preimplantation lumen diameter − postimplantation lumen diameter)/preimplantation lumen diameter. Furthermore, the expression levels of α-SMA, CD31, and eNOS were also analyzed by ImageJ software. The inflammation was scored semi-quantitatively by CD68-stained images with the following grading: 0 = no inflammation, no inflammatory cells surrounding the strut; 1 = minimal inflammation, 0 < inflammatory cells ≤5 surrounding the strut; 2 = mild inflammation, 5 < inflammatory cells ≤10 surrounding the strut; 3 = moderate inflammation, 10 < inflammatory cells ≤20 surrounding the strut; 4 = severe inflammation, inflammatory cells > 20 surrounding the strut.

### Mechanical properties of stented aorta

At 6 and 12 months postimplantation, the stented arteries were collected and maintained in NaCl solution until subsequent Young’s modulus analysis. This experiment was performed in NaCl solution with the use of a nanoindenter, which was a displacement-controlled Piuma Chiaro Nanoindenter (Optics11, Netherland, tip radius of 27.5 μm). Before measuring the samples, the cantilever (spring constant of 48.1 N/m) was submerged in NaCl solution and calibrated against a thick glass slide. After that, the indenter sensed samples and then indented in 3 μm at a loading velocity of 1 μm/s for a duration of 1 s. Last, the Young’s modulus of each sample was recorded in a 9 × 9 array with a step size of 50 μm.

### RNA-seq and data analysis

At 3 weeks post-implantation, the stented arteries were harvested. Total RNAs were extracted and quantitatively measured for quality. Then, sequencing libraries were built using the KAPA Stranded RNA-Seq Library Prep Kit (Illumina). Afterward, the RNA sequencing (RNA-seq) libraries of different samples were sequenced for 150 cycles using Illumina NovaSeq 6000 (Aksomics). The RNA-seq and analysis were conducted by Aksomics Biotech Co. Ltd. (Shanghai, China). The raw sequencing data was processed using the TruSeq SR Cluster Kit v3-cBot-HS (Illumina Inc., USA). The joint sequences and short fragments in reads were filtered to obtain clean reads and then were mapped to the reference genome using HISAT2. DEGs were identified using the Ballgown package. The threshold of│log_2_FC│ > 2, and *P* < 0.05 was set for markedly differential expression. The differentially expressed mRNAs were analyzed with the Database for Annotation Visualization and Integrated Discovery for GO enrichment analysis. The enriched biological process (BP) that had *P* < 0.05 was considered significantly enriched.

### Statistical analysis

All experiments were repeated at least three times, and the results were expressed as means ± SD. One-way analysis of variance (ANOVA; *t* test) was used to determine statistical significance between different samples undergoing the same treatment. *P < 0.05, **P < 0.01, ***P < 0.001, and ****P < 0.0001. Histomorphometric, immunohistochemical, and immunofluorescent staining results were analyzed by ImageJ software.
