# Supplementary material for: Dynamic stiffness enables stage-specific properties mediating functional endothelialization on vascular implants
Source: Sci Adv. 2026 Jul 29;12(31):eadw8744. doi: 10.1126/sciadv.adw8744 (PMC13418534; doi:10.1126/sciadv.adw8744)
Supplement: Supplementary file 1 — Figs. S1 to S21 Tables S1 and S2 Supplementary Text Legend for data S1 [file sciadv.adw8744_sm.pdf]

Science Advances
AAAS

Supplementary Materials for

Dynamic stiffness enables stage-specific properties mediating functional endothelialization on vascular implants

Li Yang *et al.*

Corresponding author: Rifang Luo, lrifang@scu.edu.cn; Yunbing Wang, yunbing.wang@scu.edu.cn

Sci. Adv. **12**, eadw8744 (2026)
DOI: 10.1126/sciadv.adw8744

The PDF file includes:

Figs. S1 to S21
Tables S1 and S2
Supplementary Text
Legend for data S1

Other Supplementary Material for this manuscript includes the following:

Data S1

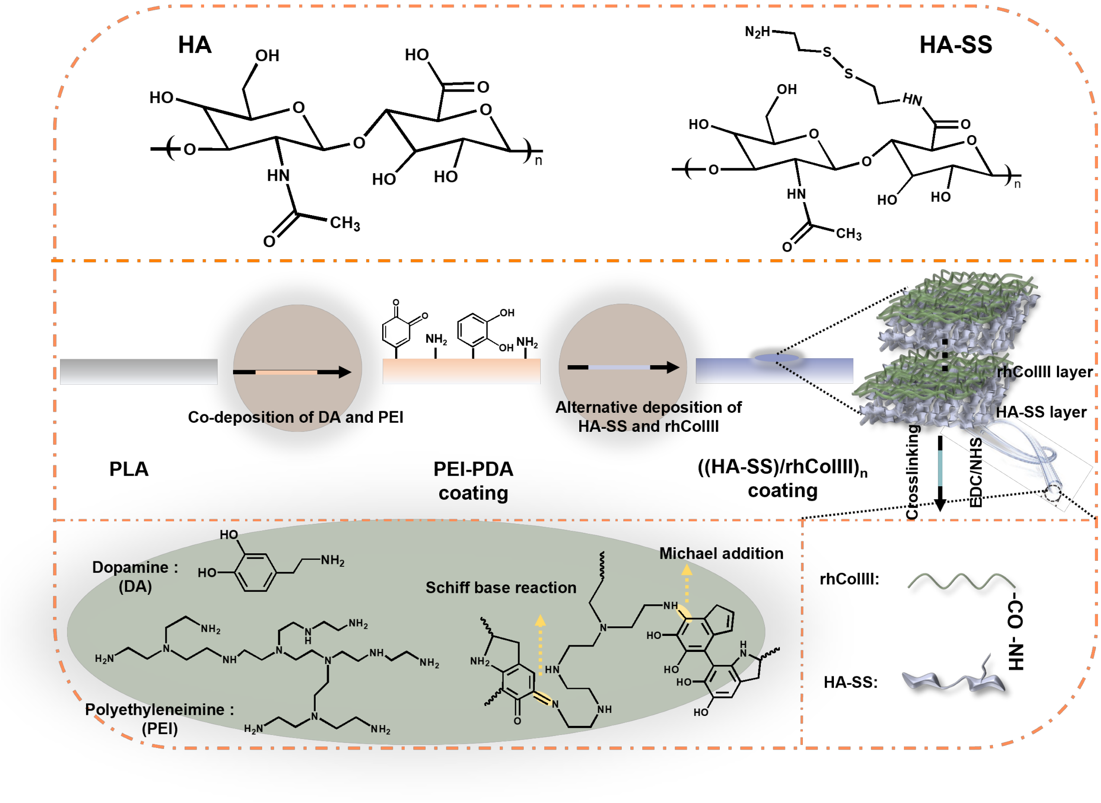

Supplementary Fig. 1 | Schematic of the ((HA-SS)/rhCol III)$_n$ fabrication process. In brief, from bare PLA to ((HA-SS)/rhCol III)$_n$-modified poly (l-lactic acid) (PLA), the preparation process initially involves amination, followed by the alternate deposition of HA-SS and rhCol III by LBL assembly technology, and finally cross-linking with EDC/NHS.

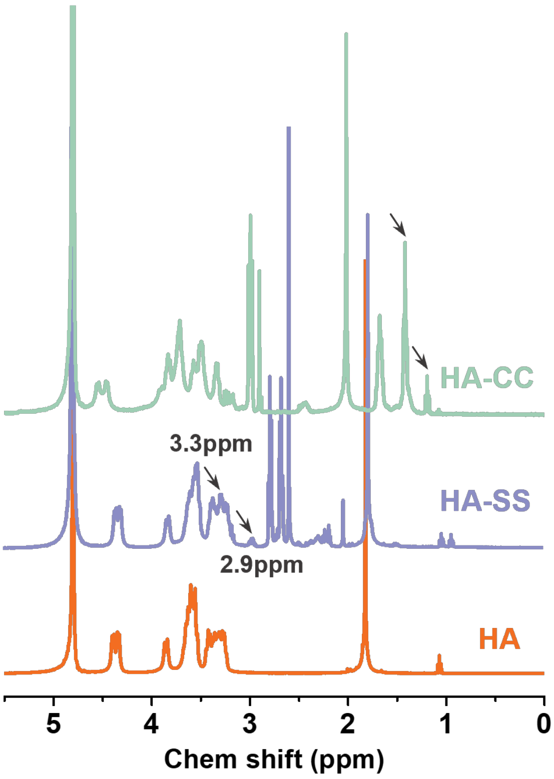

Supplementary Fig. 2 | ¹H-NMR spectra reveal structural differences among HA, hyaluronan acid-grafted Cys (HA-SS), and HA-1,6-hexanediamine (HA-CC). ¹H-NMR spectra of HA, HA-SS, andHA-CC.

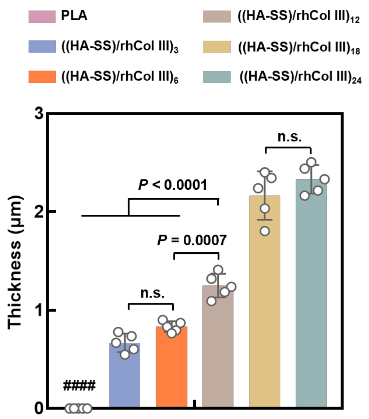

Supplementary Fig. 3 | Thickness quantification from vertical-section fluorescence images. Quantification of thickness depending on the representative vertical section fluorescent images images. n= 5. One-way ANOVA was used for the comparisons. All error bars are mean ± s.d.

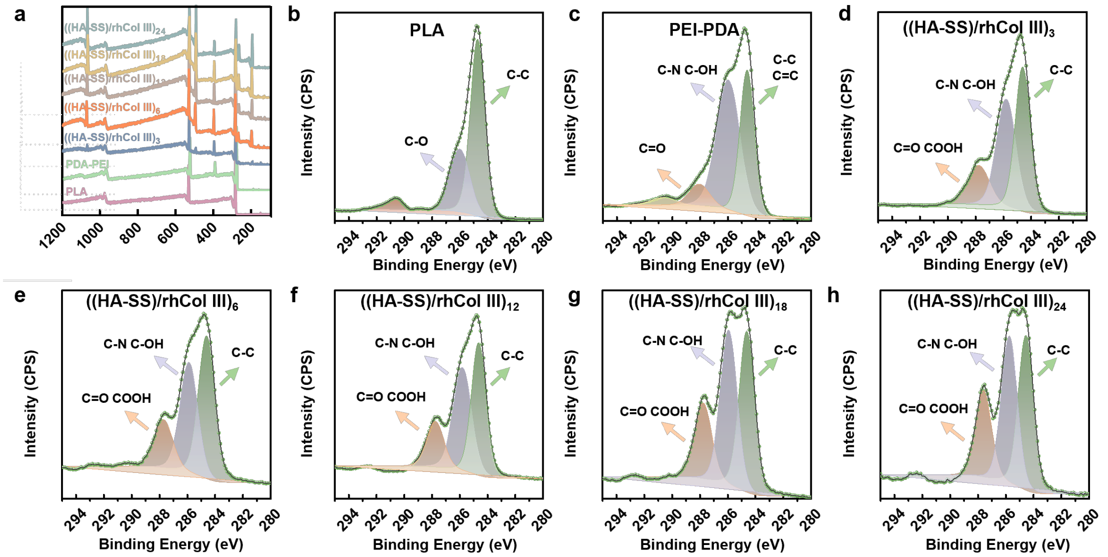

Supplementary Fig. 4 | XPS spectra show surface chemical composition of different coatings. a, Wide scan XPS spectra of uncoated, PDA-PEI- and ((HA-SS)/rhCol III)n-coated PLA sheets. **b-h**, Corresponding high resolution of the C1*s* spectrum of different samples.

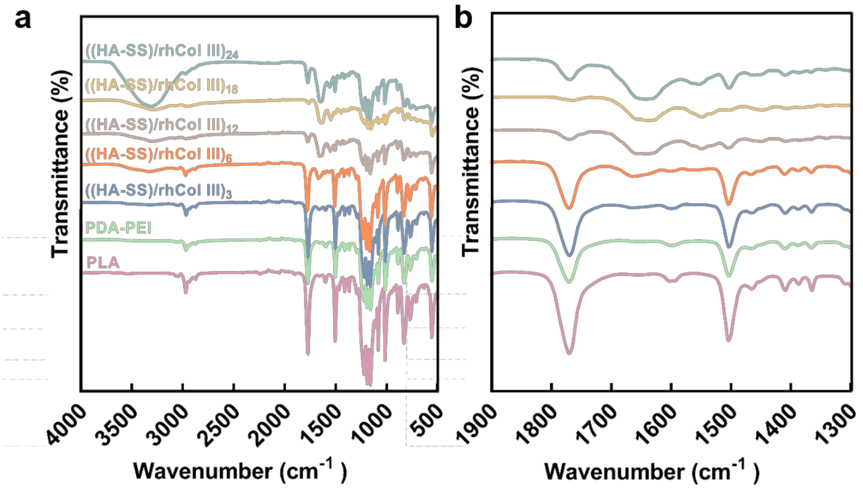

Supplementary Fig. 5 | RA-FTIR spectra confirm chemical signatures of HA-SS and rhCol III in coatings. a, RA-FTIR spectra of uncoated, PDA-PEI- and ((HA-SS)/rhCol III)n-coated PLA sheets. b, Corresponding local enlarged RA-FTIR images. The appearance of characteristic bands and peaks of HA-SS and rhCol III, assigned to O-H/N-H stretching (peaks at 3500–3200) and C=O stretching (peaks at 1680–1630 cm $^{-1}$).

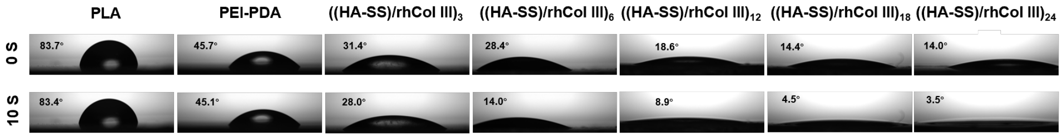

Supplementary Fig. 6 | WCA measurements indicate surface wettability changes.

Representative WCA images of uncoated, PDA-PEI- and ((HA-SS)/rhCol III)$_n$-coated PLA sheets at 0 and 10 s.

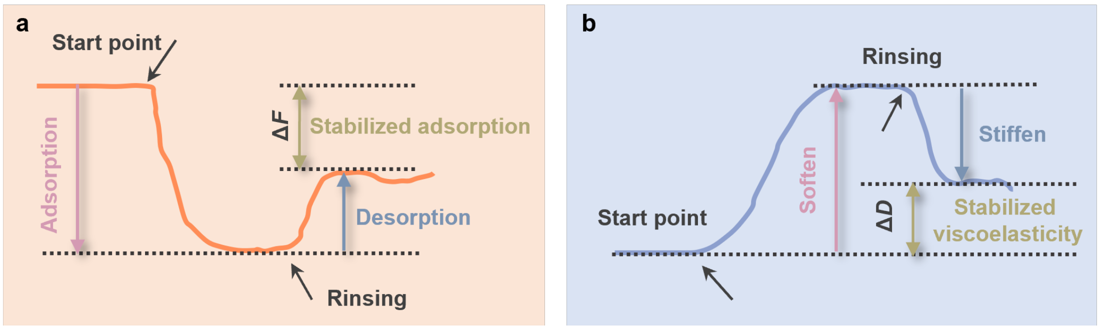

Supplementary Fig. 7 | Schematic of frequency and dissipation variation modes in QCM-D. Schematic illustration of the fundamental modes of variation in **a,** frequency and **b,** dissipation.

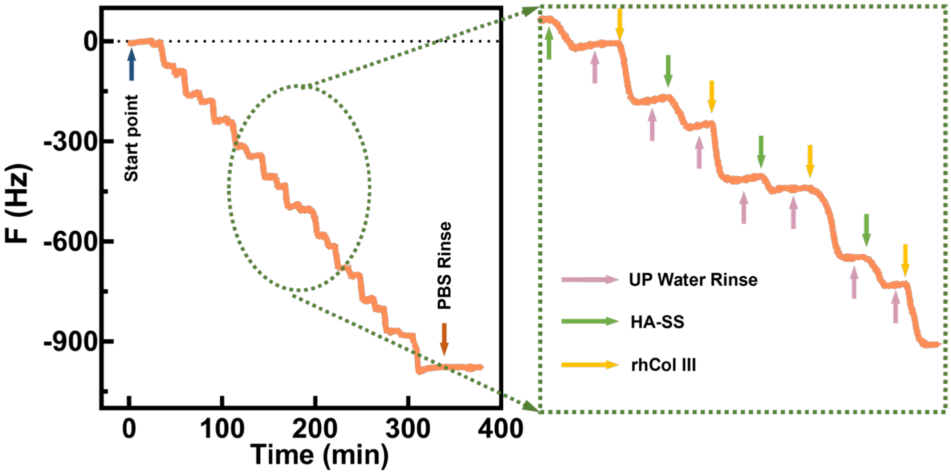

Supplementary Fig. 8 | Real-time QCM-D monitoring of F – Time during coating assembly. Representative real-time QCM-D monitoring of F-Time curve and during (HA-SS)/rhCol III assembly on PDA-PEI-modified gold slides. Regions outlined in green are expanded on the right.

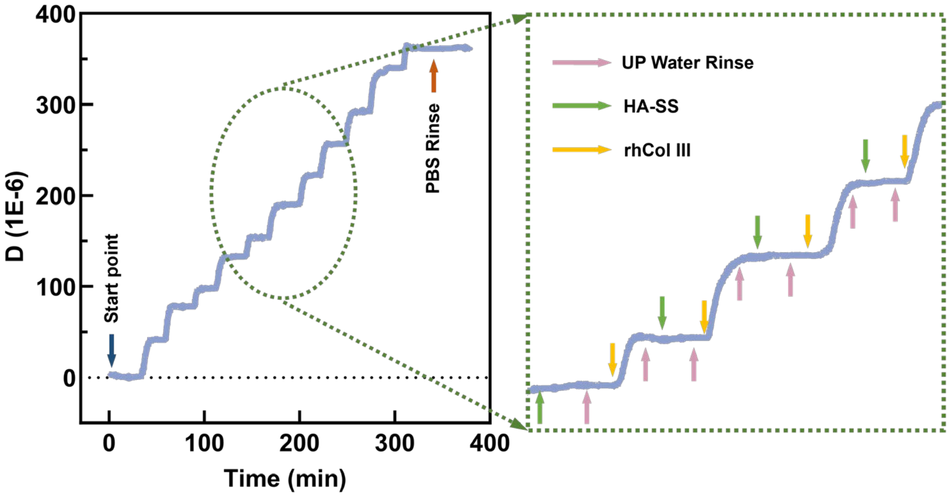

Supplementary Fig. 9 | Real-time QCM-D monitoring of D − Time during coating assembly. Representative real-time QCM-D monitoring of D-Time curve and during (HA-SS)/rhCol III assembly on PDA-PEI-modified gold slides. Regions outlined in green are expanded on the right.

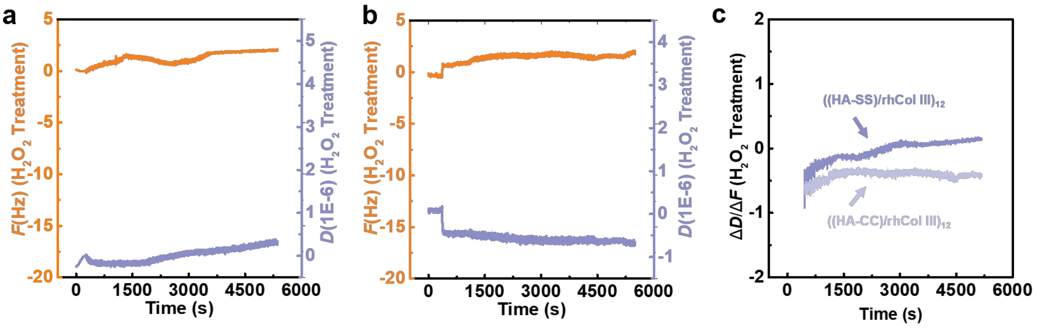

Supplementary Fig. 10 | QCM-D analysis of coating response to H_2O_2 treatment. Representative real-time QCM-D monitoring of F and D changes for **a,** ((HA-SS)/rhCol III)$_{12}$ and **b,** ((HA-CC)/rhCol III)$_{12}$ under H_2O_2 treatment. **c,** ΔD/ΔF curves of both coatings under H_2O_2 treatment.

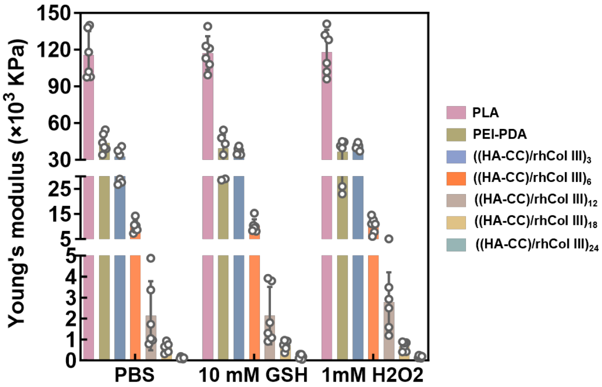

Supplementary Fig. 11 | AFM-derived Young's modulus after different treatments. Young's modulus of ((HA-CC)/rhCol III)$_n$ with PBS, GSH, and H_2O_2 treatment from AFM test.

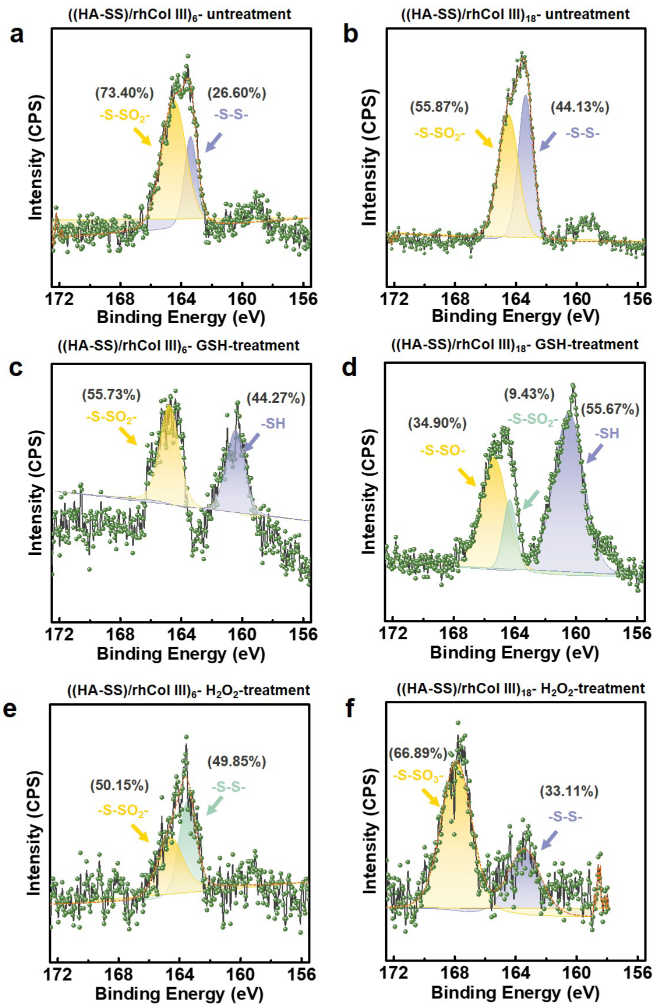

Supplementary Fig. 12 | High-resolution S2p spectra under different treatments. High resolution of the S2*p* spectrum of ((HA-SS)/rhCol III)$_6$ and ((HA-SS)/rhCol III)$_{18}$ **(a, b),** without treatment and with **(c, d),** GSH and **(e, f),** H_2O_2 treatment (n= 6 and 18).

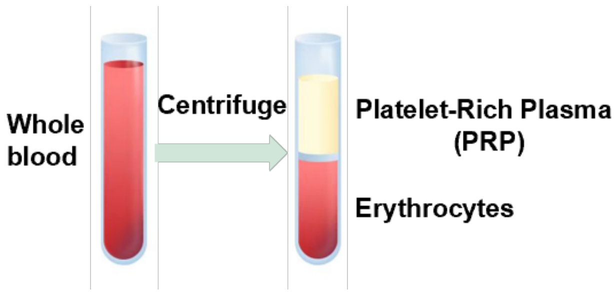

Supplementary Fig. 13 | Schematic illustration of the healthy human blood-derived Platelet-Rich Plasma (PRP) preparation.

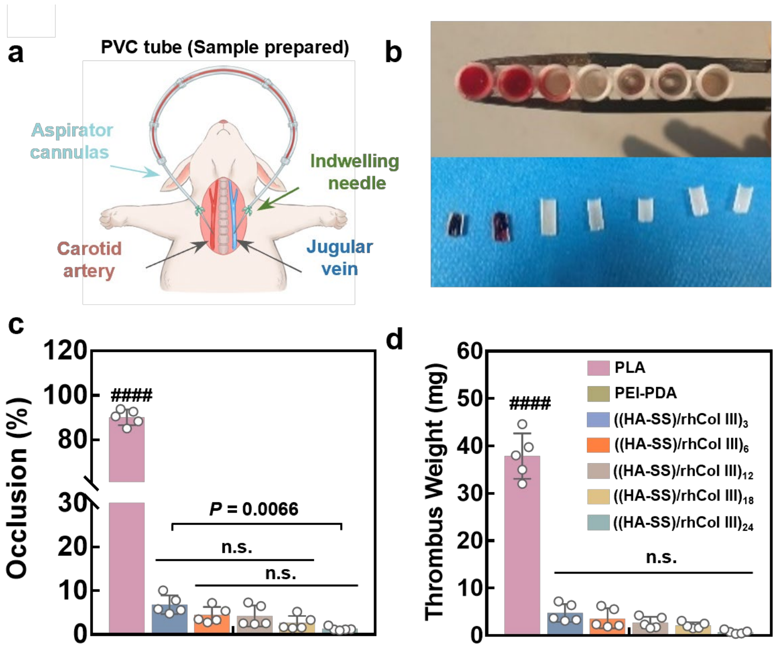

Supplementary Fig. 14 | Ex vivo arteriovenous shunt assay. a, Scheme of the New Zealand white rabbit AV shunt model exhibiting placement of the cannula in the carotid artery and jugular vein connected by the PVC tubes modified with different coatings. **b,** Cross-sectional photographs of the different PVC circuits. corresponding quantified **c,** occlusion rates and **d,** thrombus weight of the the uncoated-, PEI-PDA-coated, and $((HA\text{-}SS)/rhCol\ III)_n$-coated PVC tubes. n=5. One-way ANOVA was used in **c-d**. All error bars are mean ± s.d (P values <0.05 were considered statistically significant, #### indicated P values < 0.0001 compared with other groups).

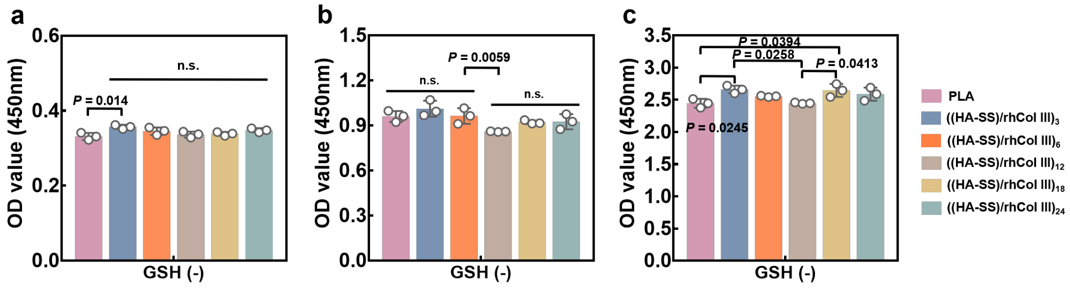

Supplementary Fig. 15 | HUVEC viability on coatings without GSH treatment. Cell viability of HUVECs cultured on uncoated and $((HA\text{-}SS)/rhCol\ III)_n$ -coated PLA sheets (n=3, 6, 12, 18 and 24) without GSH treatment after **a**, 1 day, **b**, 3 days, and **c**, 7 days of culture. n= 3. One-way ANOVA was used for the comparisons in **a-c**. All error bars are mean ± s.d (*P* values <0.05 were considered statistically significant).

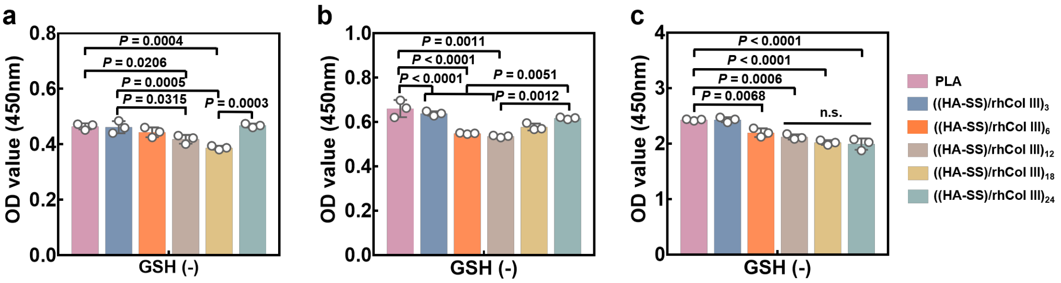

Supplementary Fig. 16 | HUASMC viability on coatings without GSH treatment. Cell viability of HUASMCs cultured on uncoated and ((HA-SS)/rhCol III)$_n$ -coated PLA sheets (n=3, 6, 12, 18 and 24) without GSH treatment after **a**, 1 day, **b**, 3 days, and **c**, 7 days of culture. n= 3. One-way ANOVA was used for the comparisons in **a-c**. All error bars are mean ± s.d (*P* values <0.05 were considered statistically significant).

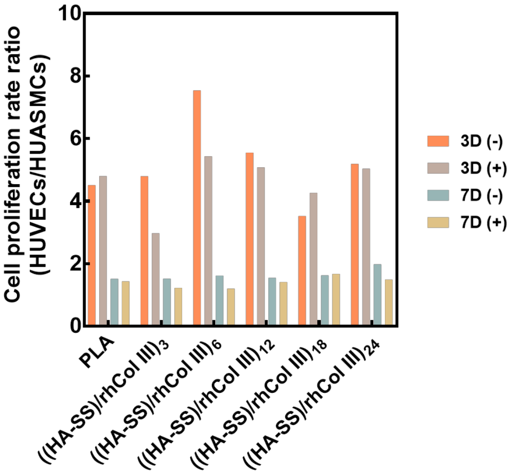

Supplementary Fig. 17 | HUVEC/HUASMC proliferation ratio with/without GSH treatment. Cell proliferation rate ratio (HUVECs/HUASMCs) of uncoated and ((HA-SS)/rhCol III)$_n$ -coated PLA sheets (n=3, 6, 12, 18 and 24) with and without GSH treatment after 3 and 7 days of culture.

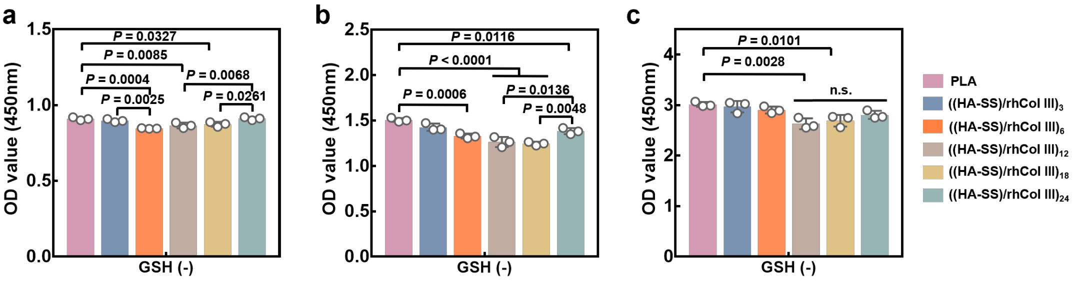

Supplementary Fig. 18 | Macrophage viability on coatings without GSH treatment. Cell viability of macrophage cultured on uncoated and ((HA-SS)/rhCol III)$_n$ -coated PLA sheets (n=3, 6, 12, 18 and 24) without GSH treatment after **a**, 1 day, **b**, 3 days, and **c**, 7 days of culture. n= 3. One-way ANOVA was used for the comparisons in **a-c**. All error bars are mean ± s.d (*P* values <0.05 were considered statistically significant).

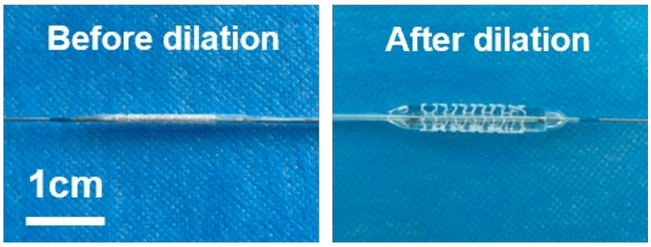

Supplementary Fig. 19 | Mechanical stability of coating after balloon dilation. Photographs of a vascular stent coated with ((HA-SS)/rhCol III)$_{18}$ before and after balloon dilation in PBS at 37 °C.

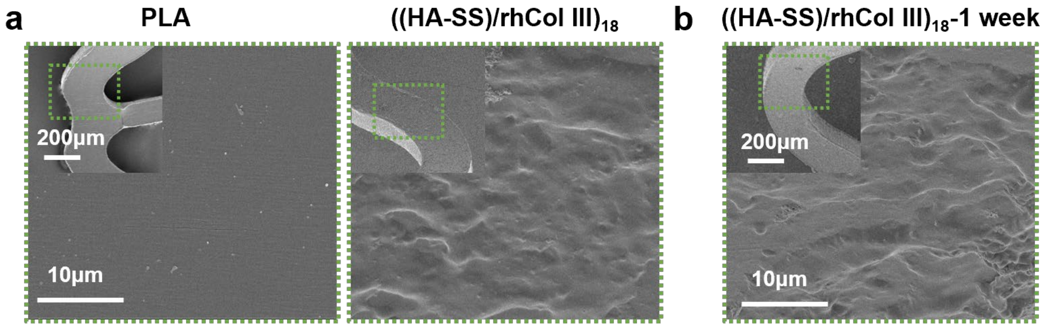

Supplementary Fig. 20 | Mechanical stability testing of the ((HA-SS)/rhCol III)$_{18}$ coating on a bare PLA stent. a Representative SEM images showing that the ((HA-SS)/rhCol III)$_{18}$ coating remained connected without cracks after balloon dilation in PBS at 37°C. Scale bars, 200 μm and 10 μm. Five samples were repeated independently with similar results. **b** Representative SEM images showing that the strut surfaces of the dilated stents coated with ((HA-SS)/rhCol III)$_{18}$ coating after 1 week of circulation under the flowing system with PBS at 37°C. Scale bars, 200 μm and 10 μm. Five samples were repeated independently with similar results.

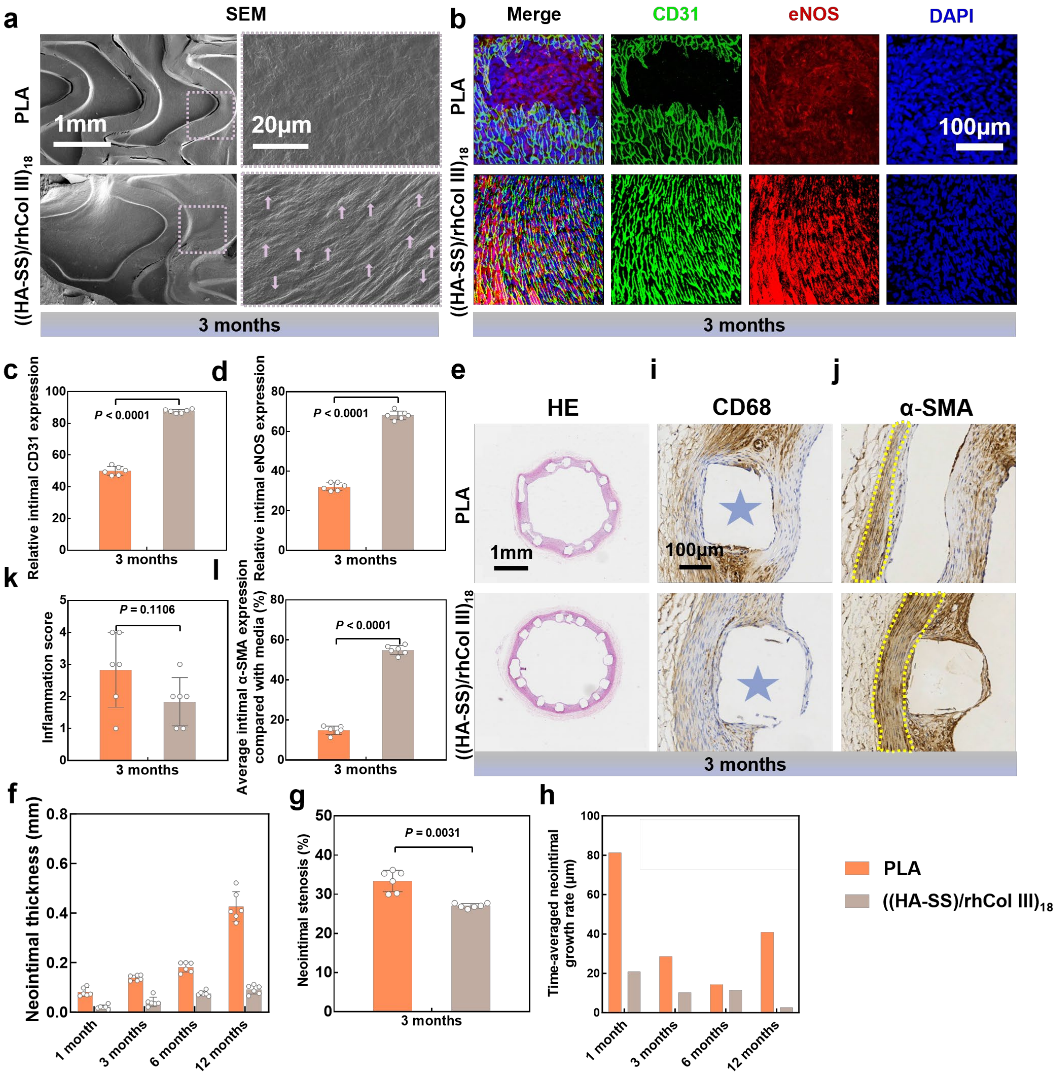

Supplementary Fig. 21 | Vascular stent placement in rabbit model for 3 months. a, Representative SEM images of luminal faces. Scale bars, 1 mm and 20 μm. Cobblestone-like endothelial cells are indicated by purple arrows; enlarged views shown below. Three independent specimens were observed with similar results. **b,** Representative CD31 (green) and eNOS (red) immunofluorescence images by CLSM; stent struts outlined in white dashed lines. Scale bars, 100 μm. Six independent specimens were observed with similar results. Corresponding quantification of **c**, the relative intimal CD31 expression, and **d**, the relative intimal eNOS expression of bare PLA and (HA-SS)/rhCol III)$_{18}$-coated PLA stents (n = 6 independent specimens from independent animals). **e,** Representative hematoxylin and eosin (HE) staining of stented arteries. Scale bars, 1mm. Corresponding quantification of **f**, neointimal thickness, **g**, In-stent lumen loss rate, **h**, time-averaged neointimal growth rate, determined from the

HE images. (n = 6 independent specimens from independent animals). Representative **i**, CD68, and **j**, α-SMA immunofluorescence staining images of stent arteries. Scale bars, 100 μm. Purple asterisks on CD68 images marked the stent struts. The media area on SMA images was outlined with yellow dashed lines. Corresponding quantification of **k**, the inflammation score centered around the strut **l**, and the α-SMA expression in the intima compared with the media (n = 6 independent specimens from independent animals). A paired t-test was used for the comparisons in **c, d, k, l,** and **g**. All error bars are mean ± s.d. (*P* values <0.05 were considered statistically significant).

Supplementary Table 1. Primers related to macrophage polarization

Primer name	Orientation	Primer sequence（5'-3')	Product length
β-Actin	FORWARD	GTGCTATGTTGCTCTAGACTTCG	174
	REVERSE	ATGCCACAGGATTCCATACC	
CD86	FORWARD	CGTTACTCTCCTGCCATCCTTC	212
	REVERSE	CATTTGTGGTGGGAGAAACTGT	
CD206	FORWARD	GAGGATATGAAGCCATGTACTCCTT	199
	REVERSE	TCGCTTCCCTCAAAGTGCAAT	
CD80	FORWARD	ACCCCCAACATAACTGAGTCT	102
	REVERSE	TTCCAACCAAGAGAAGCGAGG	
CD163	FORWARD	CTCCTGTGGACTCTGAAGCGAC	187
	REVERSE	TAGACAAAGATGTCAGTCCATCATCA	
iNOS	FORWARD	CAAGGCCACATCGGATTTCA	174
	REVERSE	TCTATTTTTGCCTCTTTAAAGGAGC	
Arginase-1	FORWARD	CTTGCGAGACGTAGACC	102
	REVERSE	ATCACCTTGCCAATCCC	
IL-1β	FORWARD	TGCCACCTTTTGACAGTGATG	220
	REVERSE	AAGGTCCACGGGAAAGACAC	

DECTIN-1	FORWARD	TAGGCCTTTCCCGCAATCAG	112
	REVERSE	TGCAGTAAGCTTTCCTGGGG	
TNF-α	FORWARD	TCAAAATTCGAGTGACAAGCCTG	245
	REVERSE	GGTATGAGATAGCAAATCGGCTG	
TGF-β	FORWARD	ACGTGGAAATCAACGGGATCAG	270
	REVERSE	GACAGAAGTTGGCATGGTAGCC	
IL-6	FORWARD	AGTTCCTCTCTGCAAGAGACTTCC	214
	REVERSE	TTGCCATTGCACAACTCTTTTC	
IL-10	FORWARD	CAACATACTGCTAACCGACTC	77
	REVERSE	GGATCATTTCCGATAAGG	

Supplementary Table 2 | Comparison of our stents with functional stent platforms, first- and second-generation DESs, and BRSs

Stent Name	Stent designs			Thrombogenicity	Endothelialization				Neointimal thickness (mm)			
	Stent base	Stent coating	Therapeutics		1 month	3 months	6 months	12 months	1 month	3 months	6 months	12 months
**I. Functional Stent Platforms**												
**(HA-SS)/rhCol III)$_{18}$-coated Stent (our data) (#)**	**PLA**	**(HA-SS)/rhCol III)$_{18}$**	**(HA-SS)/rhCol III)$_{18}$**	**Low**	**Complete**	**Complete**	**Complete**	**Complete**	**0.035 mm**	**0.041 mm**	**0.076 mm**	**0.091 mm**
As_2O_3- eluting stent(*3*)(#)	316L SS	Heparin	Arsenic trioxide	Low-Medium	Incomplete	Complete	Complete	NR	0.22 mm	0.24 mm	0.27 mm	NR
Akt1 siRNA-eluting stent(*50*)(#)	316L SS	Hyaluronic acid	Akt1 siRNA	Low-minimal	Complete	NR	NR	NR	0.39 mm	NR	NR	NR
Retinoic acid eluting stent(*69*)(#)	CoCr	PLGA or PLA	Retinoic acid	Low	Incomplete	NR	NR	NR	0.075 mm	NR	NR	NR
Cytochalasin D-eluting Stent(*70*)(&)	316L SS	PLGA	Cytochalasin D	Low	Complete	NR	NR	NR	0.17mm	NR	NR	NR
D65495-eluting stent(*71*)(#)	CoCr	None	D65495	High	Complete	Complete	NR	NR	NR	0.049 mm	NR	NR
Gene-eluting stent(*72*)(# / &)	SS	NBRP	7ND cDNA	Low	Complete	Complete	NR	NR	NR	0.154mm	NR	NR
Nitric oxide-eluting stent(*73*)(&)	316L SS	Hydrogel	Nitric oxide	Low	Complete	Complete	NR	NR	0.094mm	0.133mm	NR	NR
Corolimus-eluting stent(*74*)(&)	CoCr	omega-3 fatty acid	Corolimus	Low	NR	NR	NR	NR	NR	NR	NR	0.153 mm
miR-145-eluting stent(*9*)**(#)**	CoCr	BRP	miR-145	Low	NR	NR	NR	NR	0.105 mm	NR	NR	NR
NF-κB decoy-eluting stent(*75*)**(#)**	SS	NBRP	NF-κB	High	Complete	Complete	NR	NR	0.285 mm	NR	NR	NR

COMBO™(*76*)(*)	CoCr	anti-CD34 antibody	sirolimus	Low	Incomplete	Incomplete	Complete	Complete	0.061 mm	0.097 mm	0.132 mm	0.16 mm
**II. First-generation Drug-Eluting Stents (1st-gen DESs)**												
Cypher®(*77, 78*)(# / *)	316L SS	NBRP	Sirolimus	High	Incomplete	Incomplete	NR	NR	0.062 mm	0.146 mm	NR	NR
Taxus®(*61*)(&)	PtCr	NBRP	Paclitaxel	High	Incomplete	NR	NR	NR	0.158 mm	NR	NR	NR
**III. Second-generation Drug-Eluting Stents (2st-gen DESs)**												
Orsiro sirolimus-eluting stent(*79*)(*)	CoCr	PLA	Sirolimus	Low	NR	Complete	NR	NR	NR	0.094 mm	NR	NR
Everolimus eluting stents(*80*)(&)	CoCr	NBRP	Everolimus	Low	Incomplete	NR	NR	NR	0.089 mm	NR	NR	NR
Zotarolimus-eluting stent(*81*)(&)	CoCr	NBRP	Zotarolimus	Low	Incomplete	NR	NR	NR	0.09 mm	NR	NR	NR
Sirolimus-Eluting Stent(*82*)(&)	CoCr	NBRP	Sirolimus	Low	Incomplete	NR	NR	NR	0.09	NR	NR	NR
Coracto™ Rapamycin-eluting Stent(*83*)(*)	316L SS	PLGA	Rapamycin	Low	Incomplete	Complete	Complete	NR	NR	0.15 mm	0.17 mm	NR
Xience®(*84*)(&)	CoCr	Fluorinated	Everolimus	Low	Incomplete	Incomplete	Complete	NR	0.205 mm	NR	NR	NR
Promus Element™ EES(*85*)(&)	PtCr	PVDF-HFP+PBMA	Everolimus	Low	Complete	Complete	Complete	NR	0.33 mm	0.23mm	0.19 mm	0.12 mm
Orsiro™(*61*)(*)	CoCr	PLLA + proBIO SiC	Sirolimus	Low	Complete	NR	Complete	NR	NR	NR	0.171 mm	NR
BioMatrix Flex®22(&)	316L SS	PLA	Biolimus A9	High	Incomplete	NR	NR	NR	NR	NR	NR	NR
**IV. Bioresorbable Stents (BRSs)**												
**PLA (our control) (#)**	**PLA**	**None**	**None**	**High**	**Incomplete**	**Incomplete**	**Incomplete**	**Complete**	**0.081 mm**	**0.139 mm**	**0.174 mm**	**0.427 mm**
Absorb™ BVS(*62*)(&)	PLA	PDLLA	Everolimus	Low	Incomplete	Complete	Complete	Complete	0.227 mm	0.263 mm	0.291 mm	0.218 mm
DESolve™(*63*)(*)	PLA	None	Novolimus	Low	NR	NR	Complete	NR	NR	NR	0.2 mm	NR
Fantom™ Encore BRS(*64*)(*)	Tyrocore	None	sirolimus	Low	NR	NR	Complete	NR	NR	NR	0.057 mm	NR
Magmaris™(*65*)(&)	WE43 Mg	PLLA	Sirolimus	Low	Complete	Complete	Complete	NR	0.24 mm	0.27 mm	0.28 mm	NR
XINSORB™ BRS(*66*)(&)	PLA	PDLLA	Sirolimus	Low	Complete	Complete	Complete	NR	0.104 mm	0.205 mm	0.154 mm	NR
Firesorb™ BRS(*67*)(*)	PLA	PDLLA	Sirolimus	Low	NR	NR	NR	Complete	NR	NR	NR	0.17 mm
Zn-0.8Cu BRS(*68*)(&)	Zn-Cu	None	None	Low	Incomplete	Complete	Complete	Complete	0.128 mm	0.157mm	0.178 mm	0.163

SS: Stainless steel; *CoCr:* Cobalt chromium alloy; *PtCr:* Platinum chromium alloy; *NBRP:* Non-bioresorbalbe polymer; *NR:* Not reported; #: Stent implanted in a rabbit pathological model; &: Stent implanted in a porcine pathological model; *: Stent implanted in a human patient model.

[orange] Property inferior to that of our (HA-SS)/rhCol III)$_{18}$-coated stent

[green] Property comparable to that of our (HA-SS)/rhCol III)$_{18}$-coated stent

[yellow] Property superior to that of our (HA-SS)/rhCol III)$_{18}$-coated stent

Neointimal thickness values were either directly obtained from the original references or estimated

based on luminal stenosis rate (SR) and stent diameter (D) using the following formula:

$$t = \frac{D}{2}\left(1 - \sqrt{1 - SR}\right)$$

This equation assumes concentric neointimal growth and has been commonly applied in stent performance evaluations.

Wide scan XPS spectra and RA-FTIR spectra characterization of uncoated, PDA-PEI- and ((HA-SS)/rhCol III)$_n$-coated PLA sheets

The successful fabrication of the ((HA-SS)/rhCol III)$_n$ coatings was further confirmed by the C1*s* high-resolution spectra (Supplementary Figs. 4b-h). Compared to the PDA-PEI, the new signal of the COOH peak (the fitting peaks at 288.3 eV) were clearly observed in the ((HA-SS)/rhCol III)$_n$ groups, strongly confirming the successful grafting of HA-SS and rhCol III on the PDA-PEI(*35*). The RA-FTIR results also indicated the successful construction of the ((HA-SS)/rhCol III)$_n$ coatings (Supplementary Figs. 5a and 5b), as evidenced by the appearance of characteristic bands and peaks of HA-SS and rhCol III, assigned to O-H/N-H stretching (peaks at 3500–3200) and C=O stretching (peaks at 1680–1630 cm $^{-1}$) (*36*). These changes proved the covalent cross-linking of HA-SS through an amide reaction with amino groups on the rhCol III layer, which would happen theoretically in a water-soluble EDC/NHS solution.

In vitro and ex vivo hemocompatibility of the ((HA-SS)/rhCol III)$_n$ coatings

To evaluate the potential of our ((HA-SS)/rhCol III)n coatings to suppress clot formation, we carried out in vitro platelet adhesion assay using platelet-rich plasma (PRP) (Supplementary Fig. 13) from healthy human volunteers. After 2 h of incubation, a significant number of highly activated platelets adhered and aggregated on the surface of the PLA group, presenting flattened morphology with pseudopodia spreading. A considerable number of aggregated platelets with interwoven pseudopodia still adhered to the surface of ((HA-SS)/rhCol III)$_3$; however, with the increase in the number of layers, almost no platelets were observed on the surface of ((HA-SS)/rhCol III)$_n$ (n=12, 18, and 24) (Figs. 3d and e). Consistent with the above results, ex vivo arteriovenous

(AV) shunt assay using New Zealand white rabbit model (Supplementary Fig. 14a) further confirmed that $((HA\text{-}SS)/rhCol\ III)_n$ (n=12, 18, and 24) has excellent antithrombotic properties. Specifically, to test the antithrombotic properties under real blood conditions, the $((HA\text{-}SS)/rhCol\ III)_n$ coatings were prepared on commercial PVC tubes and then assembled into arteriovenous (AV) shunt circuits in New Zealand white rabbits. The antithrombotic abilities of different samples were evaluated by analyzing occlusion rate and thrombi developed after 2 h of *ex vivo* blood circulation. Analysis of circuit cross-sections revealed that bare PVC tubes were almost completely occluded by blood clots, while PVC tubes modified with $((HA\text{-}SS)/rhCol\ III)_3$ and $((HA\text{-}SS)/rhCol\ III)_6$ coatings reduced the occlusion rate to 5.4 ± 0.53% and 3.2 ± 0.37%, respectively. The $((HA\text{-}SS)/rhCol\ III)_n$ (n=12, 18, and 24) coatings dramatically suppressed the occlusion rate of the circuits to less than 1.2% (Supplementary Figs. 14b and c). In a similar pattern, the total weight of thrombi on the $((HA\text{-}SS)/rhCol\ III)_n$ -coated circuits (n=12, 18, and 24) (1.9 ± 0.39%, 1.7 ± 0.22% and 0.5 ± 0.19%, respectively) was remarkably reduced compared to bare PVC (40.8 ± 0.53%) (Supplementary Fig. 14d). The above results strongly confirmed the capacity of adequate amounts of rhCol III in preventing the thrombi formation(*44*).

Evaluation of intimal hyperplasia inhibition efficacy between PLA stents and $((HA\text{-}SS)/rhCol\ III)_n$-coated stents

To explore the differences between different PLA and $(HA\text{-}SS)/rhCol\ III)_{18}$ stents concerning the capacity to suppress intimal hyperplasia, the stented arteries were harvested and then stained with hematoxylin and eosin (HE) at the designated time points. As shown in Figs. 8f and g, and Supplementary Fig. 21e-h, all vascular stents were completely covered, but neointimal growth rates varied significantly between groups. After implantation for 1 month, $(HA\text{-}SS)/rhCol\ III)_{18}$-coated stents revealed remarkable inhibition of neointimal formation in comparison with bare PLA stents. With the extension of the implantation time to 3, 6, and 12 months as designed, respectively, the neointima grew rapidly on the bare PLA stent, with the neointimal thickness (NT) increasing from 81 ± 15 μm to 139 ± 10 μm, 174 ± 20 μm, and 427 ±

55 μm, and In-stent lumen loss rate (LL) increasing from 20.23 ± 4.61 % to 24.48 ± 1.81 %, 30.09 ± 2.53 %, and 50.46 ± 4.37 % (Fig. 8g and Supplementary Figs. 21f and g). In contrast, these indices of (HA-SS)/rhCol III)$_{18}$-coated stents slowly increased to 91 ± 18 μm and 24.19 ± 4.35 %, respectively, after implantation for 12 months, which was significantly smaller (P<0.0001) than those of the bare PLA stent. In addition, the time-averaged neointimal growth rate on (HA-SS)/rhCol III)$_{18}$-coated stents decreased from 21 μm month $^{-1}$ in the first month to 9 μm month $^{-1}$ in the next 11 months, whereas these values for bare PLA stent are 81 μm month $^{-1}$ and 41 μm month $^{-1}$, respectively (Supplementary Fig. 20h).

Data S1 | Raw data underlying the figures.

The Excel file contains the raw data used to generate the figures presented in this manuscript.
